# Patient-derived xenograft models in cancer therapy: technologies and applications

**DOI:** 10.1038/s41392-023-01419-2

**Published:** 2023-04-12

**Authors:** Yihan Liu, Wantao Wu, Changjing Cai, Hao Zhang, Hong Shen, Ying Han

**Affiliations:** 1 Department of Oncology, Xiangya Hospital, Central South University, Changsha, Hunan 410008 China; 2 National Clinical Research Center for Geriatric Disorders, Xiangya Hospital, Central South University, Changsha, Hunan 410008 P.R. China; 3 Department of Neurosurgery, The Second Affiliated Hospital, Chongqing Medical University, Chongqing, China

**Keywords:** Cancer models, Cancer therapy

## Abstract

Patient-derived xenograft (PDX) models, in which tumor tissues from patients are implanted into immunocompromised or humanized mice, have shown superiority in recapitulating the characteristics of cancer, such as the spatial structure of cancer and the intratumor heterogeneity of cancer. Moreover, PDX models retain the genomic features of patients across different stages, subtypes, and diversified treatment backgrounds. Optimized PDX engraftment procedures and modern technologies such as multi-omics and deep learning have enabled a more comprehensive depiction of the PDX molecular landscape and boosted the utilization of PDX models. These irreplaceable advantages make PDX models an ideal choice in cancer treatment studies, such as preclinical trials of novel drugs, validating novel drug combinations, screening drug-sensitive patients, and exploring drug resistance mechanisms. In this review, we gave an overview of the history of PDX models and the process of PDX model establishment. Subsequently, the review presents the strengths and weaknesses of PDX models and highlights the integration of novel technologies in PDX model research. Finally, we delineated the broad application of PDX models in chemotherapy, targeted therapy, immunotherapy, and other novel therapies.

## Introduction

In the realm of cancer treatment, the advent of targeted therapies and immunotherapies has greatly enriched the arsenal against cancer and provided patients with better therapeutic outcomes and milder side effects. Nonetheless, many problems have restrained improvement in the prognosis of cancer patients. First, only a small proportion of cancer patients benefit from the drugs. Therefore, robust biomarkers are necessary to accurately select the patients who will respond to or resist these therapies. However, current biomarkers and signatures are insufficient to reflect the genuine cancer status, let alone classify the patients accurately.^1^ Besides, the patients who respond to these therapies must deal with resistance and cancer recurrence.^2^ They need guidance regarding the choice of next-line therapies, which relies on the dynamic detection of the cancer status.^3^ Moreover, the remaining cancer patients are in urgent need of new pharmaceuticals. Although numerous prospective drugs against cancer have been developed and shown promising therapeutic efficacy in vitro, only a few of them have been proven safe and effective in the context of complex in vivo experiments.^4^ These problems are largely due to the lack of research tools to reveal the genuine status of cancer in real-world patients.

Oncogenesis is a dynamic process that results from many intertwined factors. During cancer progression, genetic and epigenetic aberrations lead to distinct genomic landscapes among patients, and different treatments further fuel genomic evolution and remodeling.^5^ Moreover, intratumor spatial and temporal heterogeneity added complexity to cancer, which refers to the diverse cancer cell phenotypes and different states of cancer cells within a single patient caused by cancer evolution and anti-cancer treatment selection.^6^ Besides, cancer spatial architecture is shaped by the interaction among different cell components and tissue structures, such as vascular distribution. Grasping these fundamental characteristics and recapitulating such evolutionary characteristics of human cancer is the critical prerequisite for cancer models to accurately reflect the response cancer and tackle the cancer treatment challenges.

Accordingly, animal models such as genome-edited mouse models, patient-derived organoids, and patient-derived xenografts (PDX) have been used to study cancer biology and capture the cancer landscape.^7^ Genetically engineered mice (GEM) models and GEM-derived allografts are suitable for studying the role of specific genes in cancer initiation and development. However, such models cannot reflect diverse driver mutations and extensive genomic alterations observed in human cancer. Chemical carcinogen-induced mouse models are time-honored models that serve as a tool to study cancer etiology and cancer biology. However, these models induce unpredictable cancer landscapes that are hard to repeat, let alone the effects of different dosing protocols and animal strains. Besides, in the animal-derived cancer models mentioned above, their interspecies inconsistency with human cancer leads to underlying distinct protein functions and oncogenesis mechanisms. Therefore, human cancer originated models are more reliable for studying therapies that rely on complex intracellular pathways and intercellular interactions. Human cancer cell lines and cell line-derived xenografts have been widely used because of their consistency, availability, and cost-effectiveness. Besides, the 3D culture of in vitro cancer cell lines partly mimics the in vivo structure of cancer, such as the different morphological and biological features of different cell layers. However, they only represent the cancers with specific gene aberration under a settled genomic background. Besides, these models lack tumor heterogeneity and fall short of recapitulating tumor architecture. Consequently, the drug response of these models seldom represents the authentic response of patients, and their consistency with clinical trials in drug response is limited.^8^

To faithfully reflect the landscape of human cancer, advanced preclinical models have been exploited. Microfluidic models facilitate the development of tumor-on-chip, a type of in vitro platform that accurately emulates various properties in the cancer microenvironment, such as the changes of glucose or oxygen availability at different positions in tumor structure. Such platforms enable researchers to manipulate many factors that may affect cancer growth and explore the effect of these factors on cancer.^9^ Although tumor-on-chip can emulate more and more components in the cancer microenvironment, such as microvascular structure,^10^ their consistency with cancer patients has not undergone rigorous examination. Besides, limited by technology support, tumor-on-chip has a long way to go before it gets popularized in cancer research centers worldwide. Comparatively, models that utilize samples derived from cancer patients remain the most plausible and accessible choice in reflecting genuine cancer structure. Patient-derived organoids (PDO), which are cultured from cancer cells from patients, serve as a cost-effective tool to retain cancer information. Thanks to technical support such as 3D co-culture assays, researchers seek to recapitulate cancer cell-stromal interactions in PDO models.^11^ However, in vitro models are not ideal for novel therapy testing because they cannot reflect many in vivo properties, such as pharmacokinetics performance, when it comes to preclinical drug testing. The PDX models are established by transplanting fresh tumor tissue resected from human cancer into mice.^12^ Comparatively, the PDX model excels in reflecting the characteristics of cancer and simulates tumor progression and evolution in human patients. The PDX model produces the most convincing preclinical results and is considered one of the most promising models to handle the conundrum troubling clinicians, such as identifying prognosis biomarkers, exploring the effect of intratumor heterogeneity on tumor progression, and evaluating new drugs.^13^

**Fig. 1:**
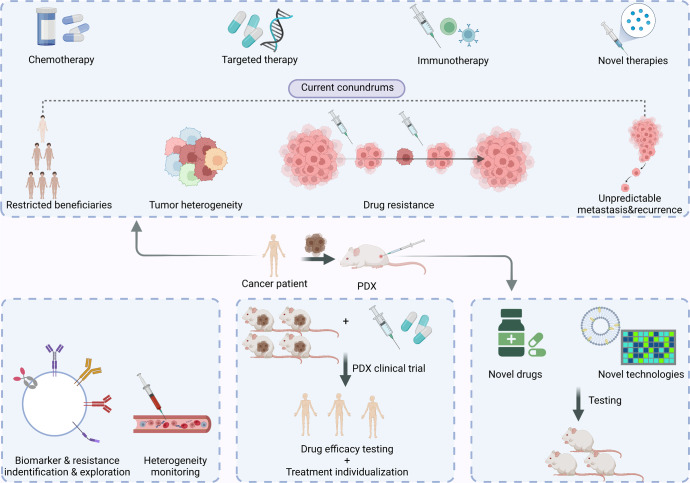
PDX in the new era of cancer treatment. This figure shows the current conundrums of cancer treatment including restricted beneficiaries, tumor heterogeneity, drug resistance as well as tumor metastasis and recurrence, and shows the versatile functions of PDX in developing therapeutics against cancer

In this review, we delineated the establishment process of PDX models and the advantages and inadequacies of PDX models. Furthermore, we summarized how the current technologies boost the application of PDX models (Fig. 1). Finally, we focused on the role of PDX in studying chemotherapy, targeted therapy, immunotherapy, as well as other novel treatments against cancer. PDX models can tackle various problems, such as testing the efficacy of novel drugs, screening drug-sensitive and drug-resistant patients, and exploring drug-resistance mechanisms. This work reviews the crucial role of PDXs in the study of cancer and sheds light on the future application of PDX models in developing therapeutics against cancer.

## A brief history of PDX models

PDX models have gone through a history of “rediscovery”. The first reported patient-derived xenograft that accorded to the definition of PDX models could be traced back to 1969 when Rygaard and Povlsen removed colon adenocarcinoma from a patient and planted the tumor fragments into nude mice.^14^ Later, researchers proved that when treated with chemotherapies, PDX models showed comparable responses as their counterpart patients.^15^ Even though discussion about the techniques of PDX model construction persisted, the unsatisfying transplantation rate of PDX models limited their application. Moreover, because researchers lacked anti-cancer drug choices back then, the function of the PDX model was confined to predicting the drug efficacy for the patient it was derived from. In contrast, more and more cancer types had in vitro cultured human cancer cell lines. Because of their consistency, cost-effectiveness, and accessibility, cell line-derived xenograft models became and have remained the workhorse of evaluating novel anti-cancer compounds.

**Fig. 2:**
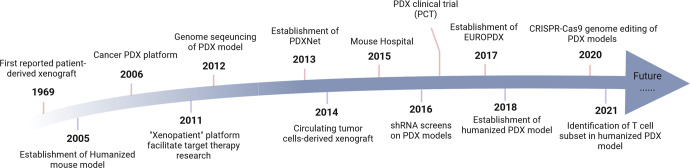
The brief timeline of milestones in PDX study

Nonetheless, researchers gradually realized the inadequacy of cancer cell lines in studying anti-cancer drug efficacy. In 2001, Johnson et al. reported that the consistency of drug response between cell-lined derived models and clinical trials was far from satisfying.^8^ The high attrition rate in pharmaceutical industries has raised dissatisfaction.^16^ In the meantime, researchers reported similar responses to chemotherapy in corresponding patients and PDX models.^17^ Moreover, researchers started to use histopathology and PCR-based technologies to validate the conformity of patients and PDX models. In 2006, Manuel Hidalgo’s group established a pancreatic PDX platform for drug screening and biomarker discovery, which was one of the pioneers.^18^ In the 2010s, the optimization of PDX establishment technologies and the popularization of sequencing technologies have boosted the resurgence of PDX models. A colorectal cancer PDX platform was utilized to identify HER-2 inhibitors to treat cetuximab-resistant patients, which is the paradigm to show the role of PDX models in targeted therapy.^19^ In 2015, Clohessy et al. developed the concept of “mouse hospital”, which refers to in vivo drug testing in models that recapitulate different cancer subtypes before heading into clinical trials.^20^ Although they mainly referred to GEM models when bringing up the concept, PDX models gradually gained wide acceptance in drug testing henceforth. In 2016, Gao established about 1000 PDX models and tested drug responses on them following clinical trial design, which is a paradigm of the patient derived clinical trial (PCT).^13^ The wide application of sequencing further validated the genomic consistency between patients and PDX models and facilitated preclinical studies of targeted therapies in PDX models. From then on, more and more platforms validated that PDX models recapitulated the cancer landscape faithfully. Therefore, in the era of immunotherapy and targeted therapy, PDX models have been widely used in preclinical drug tests. Instead of merely serving as the “avatar” of their corresponding donors, they can guide clinical decisions by predicting the molecule signatures that signify sensitivity to the drugs. A timeline highlighting the recent progress of PDX models is summarized in Fig. 2.

## PDX models: strengths and weaknesses

PDX is currently the most effective preclinical model for phenocopying cancer intratumor heterogeneity, preserving intrinsic tumor architectures, and studying drug response and resistance. Current PDX models have been applied to a wide variety of cancer types (Table 1).

**Table 1:** PDX models and biobanks of different cancer types

Reference	Cancer type (cancer subtype)	Sample number (success rate)	Factors contributing to successful PDX engraftment	Relationship between successful engraftment and patient prognosis
Hu et al.^242^	Hepatocellular carcinoma (N/A)	103 (40.6%)	Lack of encapsulation, poor tumor differentiation, large size, and overexpression of cancer stem cell biomarkers	Independent predictor for overall survival and post-resection tumor recurrence
Shin et al.^243^	Ovarian cancer (serous, clear, endometrioid, mucinous, MMMT, brenner)	61 (47%)	Tumor grade, inflammation- and immune-response-related genes	Faster PDX growth rate associated with poor prognosis of ovarian cancer and cervical cancer patients
	Cervical and vaginal cancer (squamous, adeno)	29 (64%)		
	Uterine cancer (endometrioid, serous, clear, carcinosarcoma)	18 (56%)		
Pham et al.^244^	Pancreatic cancers (ductal adenocarcinoma, mucinous adenocarcinoma, squamous, solid pseudopapillary, etc.)	118 (59%)	N/A	N/A
	Duodenal cancers (N/A)	25 (86%)		
	Biliary ductal cancers (N/A)	17 (35%)		
Kawashima et al.^245^	Acute myeloid leukemia (M0-M6, AML with MRC, tAML from MPN)	105 (66%)	N/A	Lower event-free survival rate and poor responses to chemotherapy.
Moy et al.^246^	Esophagogastric cancer (diffuse, mixed, intestinal)	98 (N/A)	Metastases, stage IV disease, HER2 expression, intestinal subtype	N/A
Jung et al.^28^	Lung squamous cell carcinoma (N/A)	82 (59%)	Tumor engraftment failure was correlated with the preoperative chemotherapy initiation.	Poor overall survival or relapse free survival.
Cybula et al.^247^	High-grade serous ovarian carcinoma (N/A)	33 (77%)	N/A	Early tumor recurrence.
Peille et al.^248^	Gastric adenocarcinoma (Intestinal, Diffuse, Mixed)	27 (27%)	Intestinal subtype	N/A
Ryu et al.^249^	Breast cancer (HR + /HER2-, HR + /HER2 + , HR-/HER2 + and triple-negative breast cancer)	20 (17.5%)	Advanced cancer stages	Poor disease-free survival, overall survival, and chemotherapy resistance
Jo et al.^250^	Lung cancer (small cell lung cancer, non-small-cell lung cancer)	55 (22%)	N/A	Chemotherapy-resistance
Xu et al.^251^	Liver cancer (hepatocellular cancer, metastatic liver cancer)	20 (38.5%)	TNM stage, lymph node metastasis, peripheral blood CA19-9 level, tumor size.	Poor median overall survival in hepatocellular cancer.
Wu et al.^252^	Malignant pleural mesothelioma (epithelioid, sarcomatoid, biphasic)	20 (40%)	N/A	Poor survival.
Bonazzi et al.^175^	Endometrial cancer (carcinosarcoma, endometrioid, mixed endometrioid, and clear cell, etc.)	18 (33%)	N/A	Shorter disease specific survival.
Strüder et al.^253^	Head and neck squamous cell carcinoma (N/A)	16 (29%)	Engraftment rate was lower and growth delayed for endoscopic biopsies.	N/A
Kamili et al.^254^	Neuroblastoma	9 (64%)	Orthotopic inoculation	Poor outcome
Miyamoto et al.^255^	Cervical cancer (Adenocarcinoma, adenosquamous carcinoma, squamous cell carcinoma)	11 (50%)	Large tumor size, high serum squamous cell carcinoma antigen and carbohydrate antigen 125 levels, and advanced FIGO stages.	Clinically poor prognoses
Schütte et al.^173^	Colorectal cancer (N/A)	59 (60%)	N/A	N/A
Tanaka et al.^256^	Acute lymphoblastic leukemia (B- acute lymphoblastic leukemia, T-acute lymphoblastic leukemia)	57 (93.3%)	N/A	N/A
Tew et al.^257^	Central nervous system metastasis (Adenocarcinoma, Squamous cell carcinoma, Invasive ductal carcinoma, etc.)	39 (84.8%)	N/A	N/A
Chapuy et al.^258^	Diffuse large B-cell lymphoma (activated B-cell (ABC)-type tumors, germinal B-cell type tumors, and plasmablastic lymphoma)	9 (32%)	N/A	N/A
Baschnagel et al.^259^	Small cell lung cancer brain metastases (Adenocarcinoma, sarcomatoid carcinoma)	9 (64%)	N/A	N/A
Elst et al.^260^	Advanced penile cancer (Usual, warty-basaloid, sarcomatoid)	11 (61%)	N/A	N/A
Lilja-Fischer et al.^261^	Oropharyngeal squamous cell carcinoma (N/A)	12 (35%)	N/A	N/A
Zhang et al.^262^	B-cell lymphoma (Mantle cell lymphoma, Burkitt’s lymphoma, Follicular lymphoma, Marginal zone lymphoma, etc.)	16 (N/A)	N/A	N/A
Chamberlain et al.^263^	Pancreatic neuroendocrine tumors (N/A)	1 (N/A)	N/A	N/A
Lin et al.^264^	Dedifferentiated endometrial carcinoma (N/A)	1 (N/A)	N/A	N/A

### PDX establishment

**Fig. 3:**
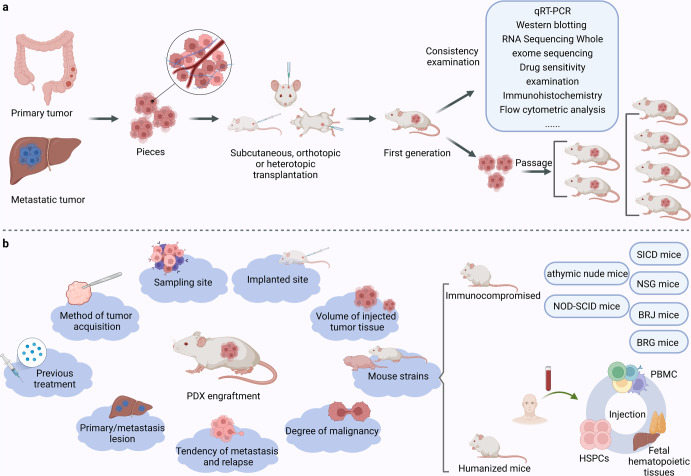
The establishment of PDX. **a** Showing the establishment process of PDX. **b** Showing the factors affecting the engraftment rate and the categories of PDX mice

To establish PDX models, primary or metastatic tumors are cut into pieces and transplanted with maintained tissue structures. The tumor pieces can be implanted subcutaneously, orthotopically, or heterotopically into the intracapsular fat pad, the anterior compartment of the eye, or under the renal capsule^12,21^ (Fig. 3). Different tumor types take different duration to establish PDX, ranging from a few days to several months. Generally, when the tumor reaches 1-2cm^3^ (first generation), it could be cut off, segmented, and reimplanted for passage. Moreover, the time of PDX establishment gradually stabilizes at 40–50 days with passages.^21,22^ To avoid tumor engraftment rejection in mouse models, conventional PDX models are typically created using immunocompromised mice, such as athymic nude mice, severe combined immunodeficiency (SCID) mice, non-obese diabetic-severe combined immunodeficiency (NOD-SCID) mice, NOD-SCID-/IL2λ-receptor null (NSG) mice, BALB/cRag2 null/ IL2λ-receptor null (BRG) mice and Rag-2 null/Jak3 null (BRJ) mice.^22,23^ Different mouse strains have various degrees of immunosuppression, and they are thus endowed with different engraftment rates, which are higher in more immunocompromised mice (BRG/BRJ > NSG > NOD/SCID > SCID > nude).^12,22,24^

PDX engraftment is affected by a series of factors. Katsiampoura et al. discovered that the method of tumor acquisition, previous treatment, and sampling site could influence the process of establishing a PDX^25^ (Fig. 3). Besides, the origin of the donor’s tumor cells, such as the primary lesion or metastasis, can affect the success rate.^26,27^ In a study of establishing lung cancer PDX models, researchers discovered that the engraftment rate hinged on the chemotherapy history of the patients.^28^ A study established a liver cancer PDX platform and discovered that NSG mice with partial hepatectomy before engraftment had better engraftment ability.^29^ In acute lymphoblastic leukemia PDX models, Richter et al. discovered that the leukemic cell subtypes could determine the site preference and growth speed of xenografts.^30^ The more aggressive high-grade and ER-negative breast cancers were found to have a higher engraftment rate.^31^ Furthermore, the engraftment of the residual tumor after neoadjuvant chemotherapy may identify a subgroup of patients with a higher risk of recurrence.^32^ Interestingly, the proportion of stroma in the tumor graft also affected the engraftment rate. Scarce stroma could lead to alimentary deficiency after tumor implantation.^33^ Besides, technical details during the transplantation process, such as the volume of injected tumor tissue, the implanted site, and the mouse strains, would contribute to the success rate.^12,33^ Despite many factors reported, the optimized protocol is still under debate and may change along with different cancer types.

In addition to the intrinsic components of the tumor microenvironment (TME), tumor-immune interaction is also implicated in cancer patient survival and tumor behaviors, which cannot be restored in general PDX. To this end, PDX with functional human immune systems could be a powerful tool for in vivo tumor immunology and immunotherapy research. Human peripheral blood mononuclear cells (PBMCs), hematopoietic stem and progenitor cells (HSPCs), or fetal hematopoietic tissues are resources for engraftment^34^ (Fig. 3). In irradiated NSG or BRG mice, human hematopoietic stem cell engraftment could lead to the development of T cells, B cells, myeloid dendritic cells, and plasmacytoid dendritic cells.^35–37^ Engraftment of human fetal liver and thymus tissue under the renal capsule of NOD-SCID mice also generated adaptive and innate immune responses.^38^ Engraftment of human PBMCs is readily available but has some remaining limitations, mainly due to the xenoreactivity of the human cells against mouse antigens. However, relatively high engraftment has been performed in BRG mice.^39,40^ Highly immunodeficient mice engrafted with functional human immune systems (more than 25% human CD45 + cells in the peripheral blood) and patient-derived tumor fragments are named humanized PDX.^41–43^

An ideal preclinical model should fully capture and maintain all the characteristics of the parental tumor and reconstruct the real tumor-immune interaction to enable in-depth insight into tumor evolution and be a silver bullet for precise drug research. Existing PDX models have shown satisfactory performance but still need to improve in several respects.

### Recapitulating parental characteristics and simulating actual tumor-immune interaction

**Fig. 4:**
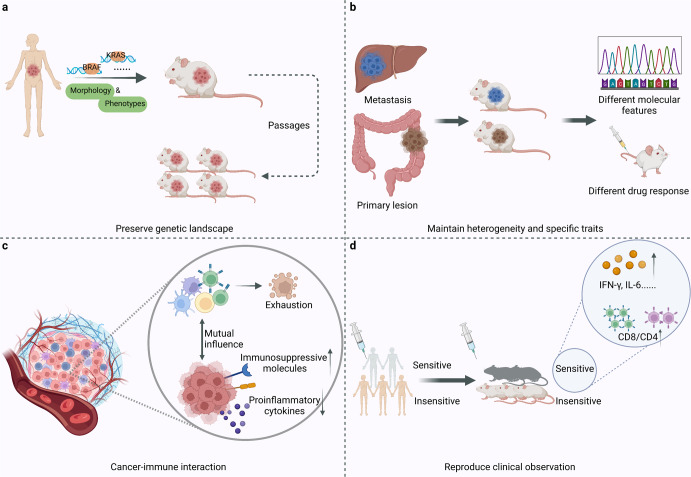
The advantages of PDX over other models. **a** PDX could preserve the genetic landscape, morphology and phenotype of the parental tumor. **b** PDX could maintain tumor heterogeneity and specific traits of metastases. **c** The realization of cancer-immune interaction in humanized PDX: human immune system tends to be educated by tumor and exhibits an exhausted status over time, and tumors maintain evolution and heterogeneity to survive with upregulated immunosuppressive molecules and decreased production of human pro-inflammatory cytokines. **d** PDX could reproduce the drug response observed in patients

Many studies have demonstrated that PDX could preserve the histopathology and genetic landscape of the parental tumor, and the clonal compositions in PDX paralleled the genetic heterogeneity.^17,44–46^ Orthotopic transplantation of glioblastoma (GBM) could retain the key phenotypes, molecular characteristics, and the similar morphology and invasion pattern.^47^ Mutation frequencies of commonly mutated genes in cancer, including KRAS, BRAF, TP53, PIK3CA, CTNNB1, and EGFR, were consistent with clinical data in PDX models.^25,48^ Zhao et al. treated patients and PDX models separately with a novel KRAS G12C inhibitor to assess the emergent genetic alteration after treatment, which was similar in both groups.^49^ PDX models can also recognize cancer subtypes based on molecular characteristics. In the context of breast cancer PDX models, Georgopoulou et al. identified 13 cellular phenotypes of breast cancer by single-cell mass cytometry. The phenotypes determined in treatment-naïve models can predict the response to anti-cancer therapies such as chemotherapies and targeted kinase inhibitors. Moreover, mass cytometry uncovered that in a single PDX model, cells with different oncogene signaling showed different responses to targeted therapies.^50^ Besides, the well-acknowledged consensus molecular subtypes (CMS) of colorectal cancer (CRC) have been recapitulated in PDX models.^51^ PDX could maintain high genomic stability through at least the first 10 passages, ensuring a long enough experiment window^52^ (Fig. 4). PDX models can also recapitulate the features of infection-related cancers. For instance, a hepatocellular carcinoma (HCC) PDX model cohort in China recapitulates HBV core antigen expression.^53^

PDX models maintain genetic profiles and intratumor heterogeneity, making them powerful tools for studying the different states among different regions in the primary lesion and metastases.^45^ For instance, Braekeveldt et al. dissected a neuroblastoma tumor from a patient and separately implanted ten tumor fragments into ten mice. Interestingly, the xenografts showed growth speed difference and transcription difference, and patient subtypes identified according to the gene clusters of these PDX models showed distinct prognoses.^54^ Wang et al. established PDX models from CRC liver metastasis and confirmed the fidelity to recapitulate the properties of their parental tumors. Despite the overall similarity, several mutations were only observed in the metastases, which may indicate the trigger of distant metastasis.^46^ Dahlmann et al. established PDX models from CRC peritoneal metastasis and characterized their molecular features and responses to targeted therapies.^55^ Cho et al. established PDX models from CRC primary lesions and metastases and compared their responses to drugs. They discovered that metastasis-derived PDX models respond to targeted therapies differently, possibly resulting from subclonal mutations acquired during tumor metastasis.^56^ Moreover, to replicate the spatial heterogeneity, Zhang et al. transplanted metastatic tumor tissue orthotopically into the same organ as the transplantation site, and the further drug efficacy test reflected the drug response of metastasis more faithfully.^57^

In addition, PDX models present an unprecedented chance to study the cancer-immune interaction in animal models. The vascular structure and antigen presentation machinery in PDX models were conserved to ensure T cell homing to the tumor and effective antigen recognition.^58^ Besides, PDX models retained MHC peptidome similarity through subsequent passages.^59^ A mutual influence relationship exists between tumors and the human immune system in clinical practice. Tumors maintain evolution and heterogeneity to survive, and immune cells are partially exhausted by tumor education. The same pattern is observed in humanized PDX. The human immune system tends to be educated by tumor. It exhibits an exhausted status over time, especially in cytotoxic immune cells and tumor-infiltrating lymphocytes (TIL) rather than peripheral T cells.^60^ Cancer cells acquired lymphocytes’ membrane immune regulatory proteins such as CTLA-4 via trogocytosis.^61^ Meanwhile, changes in tumor gene expression occur, which are reflected by upregulated immunosuppressive molecules and decreased production of human pro-inflammatory cytokines.^62^ This simulation of tumor-immune interaction in the human body allows researchers to understand better the mechanisms of tumor evolution, which was previously confined by the lack of clinical biopsies from various stages before time. One difficulty, however, is that human immune cells are not permanent in PDX because of the lack of genes encoding the cytokines necessary for human immune cells. To tackle this problem, Duy Tri Le, et al. transplanted a fresh, undisrupted piece of solid tumor into mice and obtained TIL-derived PDX.^63^ This unique PDX based on TIL in tumors does not require in vitro cell expansion and cytokine maintenance to achieve long-term immune system reconstitution. Graft versus host disease (GvHD) is another challenge that recognizes and attacks murine tissue as a foreign body, shortening the experimental window and impeding immunotherapy research. Correspondingly, many studies have generated specific PDX models with deficient MHC to eliminate GvHD.^64–66^

In addition to maintaining genomic heterogeneity, translational PDX models are supposed to reproduce the drug response observed in patients. A combination immunotherapy of nivolumab and ipilimumab significantly increased IFN-γ and IL-6 production and decreased the CD4/CD8 ratio in the PDX model, resulting in an immunoreactive TME as expected and clinically observed^60^ (Fig. 4). A plethora of studies reported the uniformity of treatment outcomes in PDX and the respective clinical data, consistent with its good mimicry of the parental tumor.^67,68^ A neoadjuvant therapy combining of 5-fluorouracil (5-FU) and radiotherapy had a concordant therapeutic effect in CRC patients and PDX.^69^ Several large-scale PDX clinical trials investigated drug responses and resistance mechanisms, and their findings confirmed the reproducibility and clinical translatability of PDX,^13,48,70^ indicating that PDX could retain therapeutic accuracy and is a functional clinically relevant model.^44^ Clinical trials aiming to determine the reliability of PDX for precision treatment, explore the mechanisms of resistance, or achieve other research objectives are summarized in Table 2.

**Table 2:** PDX related clinical trials

Cancer type	PDX type	Objective	Trial design	Phase	Current status	NCT number
Triple negative breast cancer (TNBC)	−	Determine the reliability of PDX for treatment response for individual TNBC patient	Create PDX mouse models with tissues collected pre- and post- neoadjuvant treatment	−	Completed	NCT02247037
Breast cancer	−	Explore the mechanisms of high recurrence after neoadjuvant therapy	Generate PDX and organoids from breast cancer patients with residual disease after neoadjuvant therapy	−	Recruiting	NCT04703244
Metastatic TNBC (mTNBC)	Mini-PDX	Investigate the efficacy of guided treatment based on Mini-PDX in mTNBC patients	Personalized treatment guided by mini-PDX and RNA sequencing	II	Recruiting	NCT04745975
Bladder cancer, Gastric cancer, Liver cancer, Lung cancer	−	Develop and characterize over 200 PDXs of different cancers and across different races	Tumor tissue samples of patients diagnosed with bladder cancer, lung cancer, gastric cancer or liver cancer were collected to establish PDXs	−	Recruiting	NCT04410302
Sarcoma	Nude mice	Develop a platform of PDX for soft tissue sarcomas	Establish sarcoma PDX and treat with radiotherapy and chemotherapy for translational research	−	Recruiting	NCT02910895
Prostate cancer	Mini-PDX	Guide treatment for patients resistant to abiraterone, enzalutamide or other new second-generation anti-androgenic drugs	Use the Second-generation sequencing and Mini-PDX to make personalized treatment and explore the clinical consistency	−	Recruiting	NCT03786848
Gastric cancer	zebrafish PDX	Evaluate the consistency of PDX for predicting therapeutic effect	Observe the response to neoadjuvant chemotherapy in patient and corresponding PDX	−	Not yet recruiting	NCT05616533
Head and neck squamous cell carcinoma (HNSCC)	−	Generate a biobank of PDX representing the different subgroups of HNSCC	Establish PDX with primary and recurrent tumor tissues, explore new biomarkers, novel therapy and drug resistance	−	Recruiting	NCT02572778
Breast Cancer	−	Establish a PDX platform of ER + , HER2- breast cancer	Develop novel treatment strategies and dissect signaling pathways underlying drug sensitivity and resistance	−	Completed	NCT02752893
Osteosarcoma	−	Provide patients with individualized treatment options with the help of PDX	Molecular profiling and in vivo drug testing	−	Not yet recruiting	NCT03358628
Metastatic solid tumors	Chick embryos	Use novel PDX platform to guide hyper-personalized medicine	Evaluate anti-tumor effects by ultrasound imaging and histology	−	Recruiting	NCT04602702
Metastatic non-small cell lung cancer (mNSCLC)	Humanized CD34 PDX	Comparison of clinical response and in-vivo anti-tumor response	Patients and corresponding PDXs expressing PD-L1 after failure of platinum-based combination chemotherapy will be treated with Pembrolizumab	IV	Recruiting	NCT03134456
Relapsedmantle cell lymphoma	−	Determine the feasibility of guiding personalized treatment by PDX	Patients that respond to previous treatment but experience relapse or disease progression receive treatment based on the results of the PDX	Early I	Recruiting	NCT03219047
Colorectal cancer, High-grade serous ovarian cancer, TNBC	−	Evaluate the utility of PDX as predictor to direct the use of chemo- and targeted therapies	Molecular profiling & in vivo drug testing in PDX and organoid cultures	−	Recruiting	NCT02732860
Breast Cancer	Nude mice	Develop PDX from tumor samples from surgical specimens of patients	Genetic analysis will be performed in patients who got a successful PDX	−	Recruiting	NCT04133077
Pancreatic cancer	Mini-PDX	Provide precision diagnosis and treatment for different stages of cancer patients	Generate Mini-PDX and explore the best medicine by RNA sequencing and drug sensitivity test.	−	Recruiting	NCT04373928
HNSCC	−	Develop a biobank of HNSCC PDX and guide chemotherapy	Genomic sequencing and drug sensitivity testing	−	Completed	NCT02752932
Urogenital cancer	Chick embryos	Test PDX efficiency and guide individualized treatment	Give certain medicines to PDX and determine the potentiality of each drug	−	Completed	NCT03551457

### Declining fidelity and limited representation of tumor subcluster

**Fig. 5:**
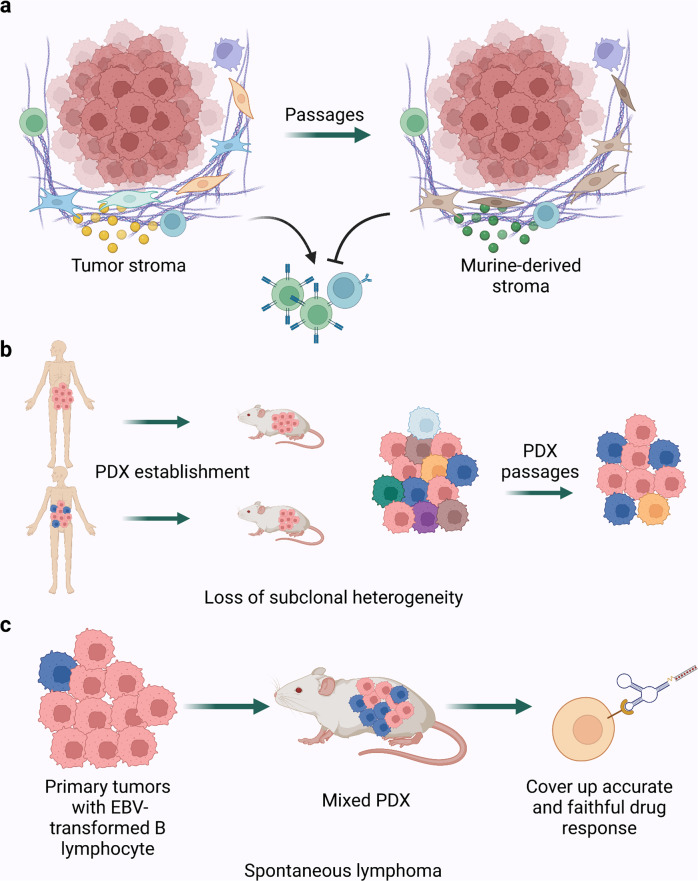
The weaknesses existing in current PDX models. **a** Tumor stroma tends to be replaced by murine-derived ECM and stromal cells after several passages, which hinders immune cell activation without human cytokine secretion. **b** Loss of subclones heterogeneity during the establishment and passage of PDX. **c** PDX mice have a relatively high risk of spontaneous lymphoma which could cover up accurate results due to the distinct drug sensitivities between lymphoma and other tumors

The tumor microenvironment represents the complexity of the tumor and its surrounding components, including the extracellular matrix (ECM), stromal cells (endothelial cells, pericytes, carcinoma-associated fibroblasts (CAFs), pro-inflammatory cells), immune cells, and secreted factors.^71^ TME provides structural support and has a massive impact on tumor development by mediating signal transduction and cell migration.^72^ Preserving TME from the donor tumor is the premise for studying tumor behavior in vivo. Studies have shown that PDX models retain principal peculiarities such as tissue structure, subtle microscopic details, and biological behaviors.^12,19,52^ However, after two to five passages, tumor stroma is almost entirely replaced by murine-derived ECM and stromal cells, among which the most predominant member, CAFs, take the fastest replacement efficiency. Even so, Arnaud Blomme et al. conducted a comparative metabolic analysis between parental tumors and corresponding PDXs. They found that metabolic profiles of both tumor cells and stromal cells remained stable for at least four passages, while the replacement occurred at the second passage.^73^ This study indicated that replacing human stroma was an acceptable drawback at the early stage of PDX research. Nevertheless, it causes trouble for the study time window and brings uncertainty for various kinds of research. Considering the critical role of CAFs in the modulation of TME, co-implantation of matched human CAFs and tumor fragments along with PDX passage may help alleviate this problem. Besides, due to species specificity, murine-derived cytokines and chemokines fail to maintain the functions of immune cells in humanized PDX. Murine IL-2 has a low activation effect on human T cells, as does murine IL-15 on NK cells.^74,75^ It hinders immune cell activation and tumor-immune interaction without human cytokine and chemokine secretion^71,76^ (Fig. 5). While periodic injections of IL-15 could maintain NK cell viability,^77^ more advanced strategies, such as genetically modified mice expressing human cytokines or growth factors, would preserve the immune microenvironment to the utmost. Anthony Rongvaux and colleagues knocked in four genes encoding human macrophage colony-stimulating factor (M-CSF), interleukin-3 (IL-3), granulocyte-macrophage colony-stimulating factor (GM-CSF), and thrombopoietin (TPO) to their respective mouse loci to support the development of human innate immune cells, including monocytes/macrophages and NK cells, which resembled the infiltration patterns in human tumors.^78^ Meraz et al. developed humanized PDX from fresh cord blood CD34 + stem cells, which reconstituted an human innate and adaptive immune system with less time and replicated human response to anti-PD-1.^41^ An updated study introduced human thymus engineering derived from inducible pluripotent stem cells to generate diverse human T cell populations in humanized mice.^79^ This novel technology may leapfrog the troubles in the scarce resources of transplanted tissues and the experimental ethics. Looking at stroma replacement another way, the composition and proportion of stromal cells could make a difference to the subtype classification of tumors, especially in CRC, thereby influencing the treatment choice. CMS4, the molecular subtype with the worst prognosis in CRC, has long been thought to have the property of epithelial-to-mesenchymal transition, which was now found to attribute mostly to stromal cells rather than tumor cells themselves.^80,81^ It reminds us that deciphering tumor cell-specific peculiarities with less stroma influence may reduce the heterogeneity of detection results to a certain extent and bring some new ideas to tumor subtype classification in clinical practice. Claudio Isella and colleagues assessed tumor intrinsic transcriptional features through human-specific expression profiling of CRC PDX and proposed CRC intrinsic gene signatures (CRIS) that exclude stroma-derived genes. Compared to CMS, CRIS more accurately reflects intrinsic tumor characteristics to stratify CRC populations and provides a better spatial and temporal classification of CRC. In addition, there is little overlap between CRIS and CMS, providing a better insight into CRC heterogeneity when used together.^82^

As mentioned above, PDX is regarded as the optimal model for describing tumor landscape. However, studies revealed that PDX underrepresented the subclonal heterogeneity, which may be critical for drug screening, while most clonal mutations could be preserved.^83^ This may be due to the sampling bias caused by spatial heterogeneity, different capacity to engraft and proliferate once injected, or tumor evolution and selection during PDX passages. In GBM PDX, although common molecular drivers were captured at frequencies comparable with the primary tumor, some alterations were gained or lost as if it were a process of clonal selection.^47^ Moreover, as GBM PDX passages increased, accelerated cell growth and increased malignancy were observed.^84^ Later-passage PDX models showed reduced similarity with primary tumors in DNA-based copy number profiles.^85^ These observations indicate that utilizing PDX with fewer passages could do better for fidelity. As for presenting the subtype heterogeneity of CRC patients, the four CMS subtypes displayed various engraftment rates and passage rates in PDX, among which CMS1 and CMS4 showed significant advantages. Given the potential subtype-specific drug sensitivity, it may skew the results of drug screening research.^86,87^ In addition, microsatellite instable (MSI) tumors carrying germline mutations easily retained their histological features compared to those with MLH1 promoter hypermethylation.^88^ Regarding intratumor heterogeneity, subclones may have different chances to keep going in PDX passages. Mixed or spindle cell uveal melanoma was characterized as epithelioid uveal melanoma in PDX, which indicates that these tumor cells were more likely to survive and grow in PDX.^33,89^ By comparing the gene signature of cancer cell subtypes between the biopsy from patients and the PDX models of small-cell lung cancer (SCLC), Lissa et al. uncovered that SCLC PDX models had a significantly higher proportion of neuroendocrine cells compared to that of the biopsy.^90^ This ill-recapitulation of intratumor heterogeneity and tumor differentiation may result from clonal selection under experimental processing or tumor evolution due to TME instability. These studies remind us that tumor heterogeneity might not be fully reflected during the establishment and passage of PDX (Fig. 5). Considering the effect of some subclones with specific biological peculiarities or drug resistance, studies such as those regarding targeted therapy could fall victim to unexpected bias. Considering that tumor heterogeneity is not only an autologous characteristic but also regulated by TME, the replacement of tumor stroma in PDX and the unstable immune system in humanized PDX may contribute mainly to the underrepresented intratumor heterogeneity through passages. Therefore, the reconstitution and monitoring of tumor stroma and the functional human immune system are essential to better maintain the tumor heterogeneity in PDX and better serve translational medicine.

Besides, the selection of mouse strains partly determines the recapitulation of cancer. The characteristics of immune system components, the engraftment rate reported by previous studies, the tendency to develop metastasis, and the susceptibility to different diseases are all key points to better match the research objectives.^22,91^ PDX mice have a relatively high risk of spontaneous lymphoma (Fig. 5). The EBV-transformed B lymphocyte in primary tumors often outgrew xenotransplantation soon after in CRC and pancreatic ductal adenocarcinoma (PDAC) PDX, especially in NSG mice.^92^ Moreover, a mixed PDX was formed after several passages, which could obstruct accurate and faithful clinical research due to the distinct drug sensitivities between lymphoma and other tumors. Since these lymphocytes generally expressed CD45, researchers proposed that sorting cells in advance could yield pure tumor cells for xenotransplantation. However, the dissociation of cells would destroy the original structure of the tumor and have a significant influence on PDX engraftment. Ovarian cancer PDX also developed unintended lymphoma frequently. Rituximab, which suppresses human lymphoproliferation, could reduce the incidence of lymphoma in subsequent PDX passages.^93^ However, the problem persisted: the effects of these interventions on tumor grafts were still undetermined. It hence highlights the need for a rigorous strategy for the detecting tumors and subsequent passages.

## Novel support technologies for the PDX model

### Improving the efficiency of PDX establishment

PDX establishment has been time-consuming and sometimes cannot serve as drug selection guidance for the donor on time. To speed up the process, Zhang et al. developed MiniPDX, which improved the implantation procedure and shortened the period for the in vivo drug response assay.^94^ In a study to establish pancreatic ductal adenocarcinoma PDX models, the authors resected the PDX tissues incompletely after growing them to a certain volume and allowed the remains to grow continuously, which turned out to grow significantly faster than the original passage.^95^ Besides, establishing and maintaining a PDX biobank requires a daunting amount of effort, which makes it hard to perform long-term or large-scale drug screening. Accordingly, researchers generated cancer cell lines^96,97^ and organoids^98,99^ from PDXs, which were proved to retain the characteristics of the original PDX models^100^ and have similar drug responses with the original PDX models in different cancer types, such as bladder cancer^101^ and melanoma.^102^

Because of the relatively long cycle and high cost of establishing mouse PDX models, researchers started looking for alternative species to serve as more efficient preclinical platforms. Zebrafish xenograft models have become a great alternative, as they support short-cycle, large-scale ex vivo assays at a low cost.^103^ Almstedt et al. established zebrafish tumor xenografts (ZTX) using glioblastoma cell cultures derived from patients and evaluated their growth longitudinally employing neural network analysis. They discovered that compared to the corresponding mouse PDX models, zebrafish models showed similar growth, invasion, and survival tendencies.^104^ In non-small cell lung cancer, Ali et al. implanted PDX tissues into zebrafish embryos and generated drug responses like mouse PDX models and the patients. Moreover, ZTX models recapitulated the invasive characteristics of the tissues and can be used to predict lymph node metastasis.^105^ In another study, Pizon et al. used chick chorioallantoic membrane to generate breast cancer PDX models, which showed a positive correlation with the primary tumor in terms of aggressiveness and proliferation.^106^

### Establishment of PDX biobanks

To facilitate the preclinical test of novel cancer treatments on PDX models, the U.S. and Europe have separately established two multi-center pan-cancer PDX consortiums, PDXNet^107^ and EurOPDX.^108^ A major challenge of multi-center PDX collection is to guarantee consistency among the PDXs from different centers. The PDXNet treated pre-validated PDX models from different centers with temozolomide, and they discovered that the PDXs from each center had the predicted response to the drug.^109^ To facilitate the management of PDX models among different centers and guarantee the quality and accessibility of the PDX models, Meehan et al. presented PDX-MI, which stands for “PDX models minimal information standard”. PDX-MI includes information regarding four aspects: clinical features, model creation, model quality assurance, and study of the model.^110^ Besides the consortium-led PDX biobanks, many organizations provide PDX model platforms with genomic data.^111^ When testing the function of novel drugs, researchers can pick out PDX models from the biobanks according to cancer types or molecular features (Table 3). As the PDX transplantation technologies become mature and the sequencing technologies accessible, researchers can tailor specialized PDX biobanks to capture the various molecular features of patients across different cancer stages, molecular subtypes, and anatomic sites. As for colorectal cancer, Mullins et al. established a PDX biobank from 149 patients with different staging, clinicopathological, and molecular features.^112^ Corso et al. established a PDX biobank of gastric cancer, which highlighted the MSI signature.^113^ To make these models more accessible, Conte et al. established the PDX Finder to help extract the characteristics of these PDX models.^114^

**Table 3:** PDX biobanks

Affiliation	Reference/website	Number of PDX cases	Cancer type
NCI Patient-Derived Models Repository	Patient-Derived Models Repository (PDMR) (cancer.gov)	Over 1000	Pan-cancer
Princess Marget Living Biobank	Princess Margaret Living Biobank | UHN (uhnresearch.ca)	Over 850	Pan-cancer
The Center for Patient Derived Models at Dana Farber Cancer Institute	Center for Patient Derived Models (CPDM) - Dana-Farber Cancer Institute | Boston, MA	Over 700	Pan-cancer
Candiolo Cancer Institute	Home | Istituto di Candiolo - FPO - IRCCS	Over 700	Gastric cancer and colorectal cancer
Charles River Laboratories	Patient-Derived Xenograft: PDX Models | Charles River (criver.com)	Over 450	Pan-cancer
Washington University in St. Louis	PDXdb: Washington University PDX Development and Trial Center | PDXdb (wustl.edu)	Over 300	Pan-cancer
Pediatric Preclinical In Vivo Testing Consortium	Pediatric Preclinical In Vivo Testing Consortium (PIVOT) – Advancing treatment options for children with cancer (preclinicalpivot.org)	Over 250	Pediatric tumors
St. Jude Children’s Research Hospital	Home | Childhood Solid Tumor Network (CSTN) Data Portal | St. Jude Cloud (stjude.cloud)	Over 150	Pediatric solid tumors
Vall d’Hebron Institute of Oncology	Home - VHIO	Over 70	Breast carcinoma, pancreas cancer, colorectal cancer
Luxembourg Institute of Health	PRECISION-PDX »Luxembourg Institute of Health (lih.lu)	Over 40	Glioma
J-PDX	^265^	Over 290	Pan-cancer

### Dynamic detection of patient status

PDX cannot fully copy the cancer microenvironment in the human body because of many innate factors, such as the surrounding murine-derived stromal cells and the lack of a fully equipped immune system. Besides, each PDX model can only reflect the status of cancer at one single stage. When a patient undergoes several lines of treatment, real-time detection is necessary to reveal the change in tumor characteristics. The detection of circulating tumor DNA (ctDNA) and circulating tumor cells (CTC) derived from liquid biopsies has been proven as an effective non-invasive method to capture the properties of cancer. Yaegashi et al. validated that if choosing the mutation targets correctly, ctDNA monitoring is sufficient to reflect the tumor burden.^115^ Cayrefourcq et al. examined the gene expression profiles of CTC lines from a patient with metastatic colon cancer, and they identified the gene dysregulations that lead to drug resistance.^116^ Therefore, in the context of “co-clinical trials” of PDXs and patients, researchers can collect more comprehensive data with patients’ non-invasive liquid biopsy results as complements. To investigate acquired resistance against targeted therapy, Russo et al. conducted a co-clinical trial and did NGS analysis on gDNA from PDX and ctDNA collected during treatment.^117^ Moreover, CTC-derived xenografts (CDX) can assist clinicians in identifying the genomic and transcriptomic features of metastatic cancer cells and guiding the treatment against late-stage cancer.^118^ Faugeroux et al. established a CDX model in the context of prostate cancer and identified that a subclone with TP53 loss triggers cancer metastasis, facilitating the drug screening for patients.^119^

### Functional genomics and PDX models

Functional genomics approaches such as the short hairpin RNA (shRNA) library and the CRISPR screen have enabled researchers to identify novel drug targets in cancer, which has been extensively applied in vitro. However, due to the lack of interaction with other cells, such experiments cannot accurately reflect cancer cell status. Therefore, when combined with PDX models, functional genomics approaches can fully uncover cancer cell vulnerabilities in the context of other cell components in the cancer microenvironment.^120,121^ Using CRISPR-Cas screening technologies, Lin et al. identified several targets against acute myeloid leukemia in PDX models.^122^ Hulton et al. developed a Cas9 lentiviral vector that can directly target PDX cancer cells in vivo, which significantly facilitates the gene programming of PDX models and the in vivo detection of potential druggable candidates.^123^ Similarly, Wirth et al. developed a chemotherapy-resistant acute lymphoblastic leukemia PDX model and conducted in vivo CRISPR/Cas9 dropout screens to determine the genes that cancer cells depend on. They identified BCL2 and successfully recovered the tumor sensitivity toward chemotherapy.^124^ Interestingly, Carlet et al. used Cre-ERT2 inducible RNAi to mimic anti-cancer therapy and silence genes in PDX models, which combined the properties of the Cre-loxP system and RNAi techniques.^125^ A DNA barcode sequence is a type of unique sequence which can track the exact cells or components that carry it.^126^ Researchers have loaded DNA barcodes and drugs in nanoparticles and injected them into tumor-bearing mice, which helped identify the exact drugs that most efficiently kill cancer cells.^127^

### Multi-omics and PDX models

Recent studies have emphasized the role of epigenetic changes in cancer drug resistance, which makes it necessary to detect gene modification events alongside gene expression. Multi-omics studies consist of genomics, epigenomics, transcriptomics, proteomics, and metabolomics, a thriving field providing researchers with much more data than ever before. Combining PDXs with technologies such as next-generation sequencing, transcriptome sequencing, and mass spectrometry (MS) can track the change in cancer cell status from different levels as they receive different treatments. Roche et al. compared the transcriptomics of primary tumor samples and PDX samples, and they showed consistency in the gene changes connected to cancer growth and proliferation.^128^ Moreover, the transcriptome can reflect the interaction among different components of cancer, such as the signaling transduction process between cancer cells and stromal cells.^129^ Mirhadi et al. conducted proteome analysis on a cohort of 137 NSCLC PDX models and identified different proteome subtypes with distinct outcomes and candidate targets.^130^ A study conducted metabolomic profiling in the context of PDX models of PDAC. It established a metabolic signature that significantly correlated with the prognosis of PDAC patients and aligned with the PDAC transcriptomic phenotypes.^131^ As demonstrated above, getting hold of the genome, transcriptome, proteome, and metabolome from the PDX cohort would provide numerous information that can guide personalized medicine. Moreover, the omics study’s ‘resolution ratio’ has progressed from bulk to single-cell analysis.^132^ Dimitrov-Markov et al. created PDX models of metastatic PDAC and sequenced single-cell RNA from circulating tumor cells. They found that the CTCs are highly metastatic and different from the matched primary and metastatic tumors.^133^ In another study, Mori et al. established PDX models of breast cancer that initially resided in the same breast tumors but had distinct responses to estrogen.^134^ Grosselin et al. conducted ChIP-seq at single-cell resolution to observe the chromatin landscapes of breast cancer xenografts and revealed intratumor heterogeneity at the chromatin level. They discovered that loss of chromatin marks H3K27me3, a transcriptional repressor against genes that contribute to drug resistance, was detected not only in resistant cells but also in a group of cells resident in drug-sensitive tumors.^135^ Still, researchers are developing updates on these technologies to solve new problems. The multiregional sequencing approach (MRA) collects DNA samples from multiple regions in one tumor and conducts NGS analysis to study intratumor heterogeneity. Sato et al. integrated MRA and PDX and detected the dynamic change of the subclonal architecture of CRC.^136^ Species-specific RNA sequencing has been applied to PDX models to distinguish the transcriptional signature originating from murine stromal cells and explore the crosstalk between cancer cells and stromal cells.^81^ Shared peptide allocation (SPA), a novel protein quantification method based on MS data, is designed to distinguish the unique and mutual peptides in the sample mixed with human cancer tissues and mouse tissues.^137^ The epigenome landscape reflects the epigenetic alterations of genes, which occur more frequently and diversely than genetic mutations and also reflect gene functions. DNA methylation is another common epigenetic modification form, and Tomar et al. analyzed the DNA methylome in ovarian cancer PDX models and determined the relationship between gene methylation and prognosis.^138^ The assay for Transposase Accessible Chromatin with high-throughput sequencing (ATAC-seq) identifies open chromatin regions that participate in cellular activity. ATAC-seq profile coupled with whole exome sequencing probes the active chromatin site as a potential druggable target.^139^ Interestingly, to study drug resistance in preclinical models, Tedesco et al. developed an integrated technology named scGET-seq that probes genomic and epigenomic sequences concomitantly at the single-cell level.^140^ Moreover, based on the unspliced and spliced mRNA abundance data from single-cell RNA sequencing, RNA velocity can be calculated to show the dynamic changes of the transcriptome and reflect how cancer cells go through different states.^141^ Based on mass spectrometry, the development of cytometry with time-of-flight (CyTOF) analysis supports high-throughput analysis of cell phenotypes.^50^ Meanwhile, phosphoproteomics can capture phosphoproteins, one of the most important post-translational modifications, and help to expose details about the protein status.^142^

Despite the large amount of data from the multi-omics study, it is still difficult to link the behavior of specific cancer cells to cancer progression. As mentioned above, DNA barcodes can track cellular behaviors at the single-cell level. Therefore, barcoded cancer cells can uncover the proliferation and metastasis tendency at a single-cell level, thus connecting cellular behavior with single-cell mRNA sequencing and revealing the cellular basis of intratumor heterogeneity.^143^

### Massive data analysis and PDX model

Robust data analysis tools facilitate the application of the PDX platform. CancerCellNet uses machine learning algorithms to assess the transcriptional fidelity of PDX models to natural tumors.^144^ Specially designed for PDX platforms, DRAP is a data analysis software designed that processes data separately according to different PDX preclinical trial designs.^145^ As mentioned above, one of the most important missions of PDX models is to identify reliable biomarkers of drug response based on pharmacogenomic datasets, that is, the pharmacologic and high-throughput sequencing profiles of PDX. Several computational platforms have been constructed to analyze preclinical pharmacogenomic data and identify robust biomarkers that predict patient drug response and prognosis.^146,147^ Machine learning is a revolutionary technology that has been widely used in the field of translational medicine. The neural network of machine learning can achieve diversified tasks based on massive data. PDX drug discovery datasets and those from cell lines and patients provide a platform with adequate data, and researchers have accomplished various tasks using this platform. Based on the multi-omics dataset in PDX studies, artificial intelligence is widely exploited to predict a patient’s response to treatment. Jiang et al. proposed that drug molecular and cellular targets, drug responses, and adverse reactions are closely intertwined. Hence, they developed DrugOrchestra, a deep learning framework that integrates the abovementioned tasks and predicts the potency of novel compounds according to their molecular structure. Besides, machine learning strategies such as transfer learning^148,149^ and few-shot learning^150^ can also address the challenge of translating the preclinical data into clinical contexts and transferring the predictors from preclinical models to human cancer applications.

### Imaging systems and PDX model

Many studies sought to test the commonalities between xenografts and tumors in the human body from the perspective of radiomics. Magnetic resonance imaging (MRI) helps to extract diverse information from PDX models. Tiwari et al. optimized MRS in a determined sequence to compare the production of 2-hydroxyglutarate between the PDX models and patients, which proved the similar IDH metabolism between PDX models and parental tumors.^151^ In the context of sarcoma PDX models, Jardim-Perassi et al. integrated the data of multiparametric MRI and histology into deep learning models to predict hypoxia and the response to hypoxia-activated prodrugs.^152^ Roy et al. extracted 48 features such as noise, resolution, and tumor volume from the MRI results of triple-negative breast cancer PDX models and discussed how the factors affect the radiomic analysis.^153^ Following this study, they further identified 64 robust features and used machine learning to select several strong biomarkers that constituted signatures that can be used to predict prognosis.^154^

Besides, integrating imaging technologies and PDX models contributes to the study of medicine uptake and distribution in a non-invasive manner. Using micro-computed tomography (µCT) imaging on PDX models, Moss et al. clarified that the density of vessels supporting pericytes is critical to liposome accumulation and distribution.^155^ In another study, Russel et al. used a radiotracer named 18F-FAC, which has a similar structure to gemcitabine, to track gemcitabine uptake in pancreatic cancer PDX models. Such a surrogate helps to determine drug uptake in patients conveniently.^156^ Interestingly, Almstedt et al. tagged the patient-derived GBM cells with green fluorescent protein (GFP), implanted them in zebrafish embryos, and observed their growth by light-sheet imaging. In this manner, they realized real-time observation of cancer initiation and development.^104^

Similarly, other practical techniques, such as in vivo imaging systems, need thorough validation before they are used on patients, and the PDX model is an ideal preclinical model. In the context of ovarian carcinoma, CD24 is highly expressed. Kleinmanns et al. conjugated an anti-CD24 antibody with Alexa Fluor 750 and used the antibody to provide real-time feedback on surgeries of metastatic ovarian carcinoma PDX models^157^; while binding an anti-CD24 antibody with Alexa Fluor 680 enabled monitoring the therapeutic efficacy of carboplatin-paclitaxel against ovarian cancer in the xenografts.^158^ Fonnes et al. established an orthotopic PDX model of endometrial carcinoma in another study. They utilized fluorescence-binding antibodies against epithelial cell adhesion molecules to track cancer’s development and anti-cancer treatment efficacy successfully.^159^

## Application of PDX model in cancer therapy

Because of the PDX model’s fidelity to replicate patient diversity and the PDX model’s diversity to reflect diversified patients and real-world scenarios, the PDX model has been widely employed in exploring the pharmacokinetics and pharmacodynamics,^160^ therapeutic effects, and drug resistance mechanisms of current treatments against cancer.^161^ This part delineates the application of PDX in the field of cancer treatment, including chemotherapy, targeted therapy, immunotherapy, and other novel therapies.

## PDX model in chemotherapy research

Chemotherapy is the most classical antitumor drug and clinically verified repeatedly in various cancers. However, chemotherapy resistance is the leading cause of tumor-related death worldwide. Moreover, there is a lack of appropriate methods to evaluate chemosensitivity to guide first-line chemotherapy. The PDX model has much to do with the selection and improvement of chemotherapy, including detecting sensitivity, exploring resistance-associated characteristics, and providing a suitable platform to test novel drugs and delivery methods.

### Exploring chemosensitivity and resistance-related targets for personalized treatment

Clinicians have established a series of standards for chemotherapy selections. However, when faced with several drugs to choose from and inter-tumor heterogeneity among patients, inappropriate selection may not maximize patient benefit. With the help of PDX, researchers could test drug sensitivity at an individual level beforehand. Studies revealed that PDX-guided chemotherapy significantly upregulated overall survival (OS) and disease-free survival (DFS) compared with standard treatment of gemcitabine and oxaliplatin in gallbladder cancer patients.^162^ Moreover, for tumors responsive to polychemotherapy, such as triple-negative breast cancer (TNBC), using PDX to test the antitumor activity of a single drug and combination therapy is of great significance to guide individualized treatment with minimum toxicity.

PDX is also a practical tool to excavate targets for drug resistance and test combined drugs afterward. Researchers detected constitutive phosphorylation of spleen tyrosine kinase (SYK) in infant acute lymphoblastic leukemia PDX, the combination of SYK inhibitor and chemotherapy could significantly enhance therapeutic efficacy.^163^ Nonetheless, they also found RAS-mediated resistance to SYK inhibition, which indicates the complexity of the whole genome picture and emphasizes the importance of personalized detection and treatment. Li et al. established chemo-resistant and chemo-sensitive ovarian cancer PDX models with paclitaxel and carboplatin.^164^ Through whole exome and RNA sequencing, they screened out the most resistance-related gene HLADPA1 and validated its association with resistance to initial chemotherapy in TCGA datasets. This research method was closely related to clinical practice and provided an entry point for in-depth research.

### Developing and testing novel drugs and delivery methods

Conventional chemotherapy usually acts through DNA damage, which could be evaded by tumor cell-induced cellular dormancy. Meanwhile, other factors, such as the tumor extracellular matrix, drug bioavailability, and the effect of targeting, all affect the efficacy of chemotherapy. It is an effective and convincing way to modify and detect these influencing factors by the PDX model.

Researchers invented a small molecule inhibitor called phendione that induced a DNA damage response without causing DNA breaks or allowing cellular dormancy.^165^ This inhibitor significantly suppressed tumor growth in BRAFV600E- and NRASQ61R-driven melanoma PDX, indicating a novel way to combat chemotherapy resistance and proposing a new idea of targeted chemotherapy. Some tumors, such as appendiceal mucinous carcinoma peritonei (MCP), exhibited an inadequate response to chemotherapy due to the protective effect of abundant extracellular mucus, which impeded drug delivery. Researchers developed a combination of bromelain and N-acetylcysteine to achieve mucolysis and significantly enhanced the chemotherapeutic effect in MCP PDX.^166^ Moreover, boosting the bioavailability of drugs by chemical modification is essential to improve the efficacy of chemotherapy. Modified gemcitabine, termed 4-N-stearoylGem, strongly inhibited tumor growth in pancreatic cancer PDX and surprisingly showed an antiangiogenic effect by reducing vascular endothelial growth factor receptors expression.^167^ Combining some novel delivery systems and technologies also showed a promising future for chemotherapy. In bladder cancer PDX, the nano-sized drug delivery system poly (OEGMA)-PTX@Ce6 (NPs@Ce6) combined with chemo-photodynamic therapy markedly improved tumor targeting and suppressed tumor growth up to 98%. In addition, this combined therapy was versatile and went beyond the functions of conventional chemotherapy, with the ability to upregulate oxidative phosphorylation and reactive oxygen species generation, and downregulate tumor growth, invasion, and metastasis signals.^168^

## PDX model in targeted therapy research

Despite discovering novel targets and developing novel target therapies, many cancers respond poorly to the targeted therapies, even with several drugs combined. Researchers constantly reveal novel mutations contributing to the resistance to targeted therapies.^169^ Moreover, intratumor heterogeneity restrains the therapeutic effect of these therapies, and cancer cells become liable to mutate under the pressure of targeted therapy, which further intensifies cancer heterogeneity.^170^ Therefore, the ensuing targeted strategies may have distinct effects on different cancer cell subclones, facilitating drug-resistant subclones outgrowth and an overall poor response.^171^ The PDX model presents the molecular characteristics and the complexity of the cancer landscape, which helps to give authentic feedback when treated with targeted therapies. The combination of next-generation sequencing, immunohistochemistry, and in situ hybridization captures the histopathological and molecular signatures and guides target therapy for the patients represented by PDX models.^172^ PDX models have aided in developing numerous targeted therapies for various cancer types (Table 4). This section discusses four scenarios in which the PDX models have advantages compared to traditional models.

**Table 4:** PDX model in targeted therapy research

Reference	Drug name	Drug administration method	Gene target	Cancer type	Application scenario
Schueler et al.^266^	Gefitinib	Oral gavage	EGFR	NSCLC	Drug resistance mechanism study
Zhang et al.^267^	ASK120067	Oral gavage	EGFR	NSCLC	Novel drug validation
Chew et al.^268^	AZD4547, BLU9931	oral gavage	FGFR1, FGFR2, FGFR4	breast cancer	Therapeutic target identification
Krytska et al.^269^	crizotinib	Intraperitoneal Injections	ALK	Neuroblastoma	Drug combination validation
Shattuck-Brandt et al.^270^	KRT-232	oral gavage	MDM2	Metastatic Melanoma	Therapeutic target identification
Kinsey et al.^271^	trametinib	oral gavage	MEK	Pancreatic ductal adenocarcinoma	Drug combination validation
Coussy et al.^272^	BYL-719;	oral gavage	PI3K	PIK3CA-mutated metaplastic breast cancer	Drug combination validation
	selumetinib	oral gavage	MEK		
Coussy et al.^181^	BAY80-6946;	N/A	PI3K p110α subunit	enzalutamide-resistant luminal androgen receptor triple-negative breast cancer	Therapeutic target identification
	PF-04691502;		mTOR and PI3K		
	AZD2014		mTORC1 and mTORC2		
Hsu et al.^273^	MLN0128	oral gavage	dual mTOR complex HER 2	HR + /HER2 + Breast Cancer	Drug combination validation
	trastuzumab	intraperitoneal injection			
Harris et al.^274^	pertuzumab/trastuzumab	oral gavage	HER2	Ovarian cancer	Drug combination validation
Hashimoto et al.^275^	U3-1402	intravenous	HER3	HER3 positive cancer	Novel drug validation
Reddy et al.^276^	Pan-HER	Intraperitoneal injection	Pan-HER antibody mixture against EGFR, HER2, and HER3	TNBC	Drug combination validation
Odintsov et al.^277^	GSK2849330	intraperitoneal injection	HER3	NRG1-rearranged lung adenocarcinoma	Drug combination validation
Chen et al.^278^	compound 36 l	Intraperitoneal injection	KRAS‒PDEδ	pancreatic tumor	Novel drug preclinical validation
Barrette et al.^279^	Verteporfin	Intraperitoneal injection	YAP-TEAD	Glioblastoma	Novel drug preclinical validation
Hemming et al.^280^	YKL-5–124	Intraperitoneal injection	CDK7	Leiomyosarcoma	Therapeutic target identification
Gebreyohannes et al.^193^	Avapritinib	Oral gavage	Mutated KIT	Gastrointestinal Stromal Tumors	Novel drug preclinical validation
Karalis et al.^281^	Lenvatinib	Oral gavage	Multitargeted tyrosine kinase inhibitor	Gastric cancer	Novel drug preclinical validation
Dankner et al.^282^	Dabrafenib, encorafenib	Oral gavage	BRAF	Class II BRAF mutant melanoma	Drug combination validation
	Trametinib and binimetinib	Oral gavage	MEK		
Knudsen et al.^283^	Palbociclib	Oral gavage	CDK4/6	Pancreatic cancer	Drug combination validation
	Trametinib	Oral gavage	MEK		
Zhao et al.^189^	Neratinib	Oral gavage	Neratinib with CDK4/6, mTOR, and MEK inhibitors for the treatment of HER2 + cancer.	Pan-cancer	Drug combination validation
Yao et al.^48^	Cetuximab	Intraperitoneal injection	EGFR	Colorectal Cancer	Drug combination validation
	LSN3074753	Oral gavage	RAF		
Gymnopoulos et al.^284^	TR1801-ADC	Intravenous injection	CMet	Pan-cancer	Novel drug preclinical validation
Vaisitti et al.^285^	VLS-101	Intravenous injection	ROR1	Richter syndrome	Novel drug preclinical validation
Haikala et al.^233^	HER3-DXd	Intravenous injection	HER3	EGFR mutant non-small cell lung cancer	Novel drug preclinical validation

### Screening drug sensitivity patients

Numerous PDX biobanks worldwide collect cancer across the diverse genomic and transcriptomic background. Therefore, when a novel therapy is developed, these platforms are an ideal tool for performing preclinical screening to identify signatures and biomarkers that serve as criteria to recognize patients who can benefit from the therapy. The Novartis Institutes for BioMedical Research established a PCT that followed the “one animal per model per treatment’ design and tested the response to 62 treatment strategies.^13^ Schütte et al. generated a preclinical platform to test the drug sensitivity of clinical drugs and used multi-omics data to identify new biomarkers to predict drug responses.^173^ Lindner et al. performed protein analysis on CRC PDX models and established fourteen markers distinguishing cetuximab-sensitive tumors from cetuximab-resistant tumors.^174^ Bonazzi et al. established different subtypes of endometrial cancer. Treating them with the PARP inhibitor talazoparib revealed that the molecular subtype with a high copy number is sensitive to the PARP inhibitor.^175^ The mTOR pathway is essential for gastric cancer (GC), but the mTOR inhibitor everolimus is not universally effective in treating gastric cancer. Fukamachi et al. derived cancer cell lines from diffuse-type GC PDX models and discovered they are sensitive to everolimus. Further, they found that the GC cells from PDX belong to cluster II of diffuse-type GC in clinical practice, which is characterized by chromosomally unstable (CIN) or MSI.^176^ Similarly, PDX trials can identify patients with specific mutations that lead them to resist paticular target therapy. Kemper et al. established a PDX trial from melanoma metastases and identified the BRAFV600E kinase domain as a resistance mechanism. Then they validated the efficacy of the pan-RAF dimerization inhibitor to eliminate this subclone.^177^ In high-grade serous ovarian carcinoma (HGSC), suppression of RAD51C leads to defective homologous recombination and marks sensitivity to PARP inhibitors. Nesic et al. established RAD51C promoter methylation PDX models and discovered that while homozygous RAD51C methylation indicates PARP inhibitor sensitivity, a single copy of unmethylated RAD51C causes drug resistance.^178^

Moreover, establishing PDX models for patient subtypes resistant to current treatment options helps to investigate new therapies. Many researchers have exploited the drugs selected in PDX models on patients and tested their efficacy.^179^ Afatinib, a TKI targeting HER2 and EGFR, outstood the drug sensitivity test in a PDX model derived from a CRC patient resistant to multiple lines of treatment; the patient used afatinib subsequently and achieved progression-free survival for three months.^180^ Coussy et al. used PDX models to study the therapeutic choices for patients with luminal androgen receptor triple-negative breast cancer resistant to enzalutamide and discovered significant enrichment in PIK3CA and AKT1 mutations. Consequently, mTOR and PI3K inhibitors are proven effective for these patients.^181^ Metastatic colorectal cancer patients with KRAS and BRAF mutations have poor prognoses. Therefore, Area et al. established PDX models for them and examined the vulnerability to the PARP inhibitor olaparib and the chemotherapy drug oxaliplatin. They discovered that a subset of PDX models is deficient in HR, and they are sensitive to olaparib, and such response is positively correlated with oxaliplatin vulnerability. As oxaliplatin is a common choice for such patients, this study provides a rationale for sequential treatment with oxaliplatin and olaparib.^182^ With the aid of whole exome sequencing and transcriptome sequencing, PDX cohorts would facilitate the identification of biomarkers that can predict the drug sensitivity of patients.

### Exploring mechanisms of therapeutic effect and drug resistance

Compared to normal mouse xenograft models, PDX models can be generated with tumor cells from patients who undergo resistance to certain therapies, which makes them better models for studying the mechanisms of cancer drug resistance to existing therapies and facilitating the development of novel therapeutic targets. Utilizing establishing PDX models from drug-resistant patients, researchers identified mutation sites such as MET that led to cetuximab resistance.^183^ Silic-Benussi et al. revealed that, in the context of T-cell acute lymphoblastic leukemia (T-ALL), mTOR pathway activation led to drug resistance to glucocorticoid because of an insufficient level of ROS. When treating with the mTOR inhibitor everolimus in the T-ALL PDX model, they observed an increased level of ROS accompanied by a decreased capacity of ROS scavenging and significant therapeutic effects.^184^ Zhang et al. treated a KRAS G13D mutant CRC PDX model with cetuximab and detected the expression level changes of different genes, thereby determining potential genes contributing to acquired resistance.^185^ Another study investigated the mechanism of EGFR inhibitor resistance and discovered that the remaining tumor cells after EGFR treatment have high HER-2 and HER-3 activity. Subsequently, pan-EGFR antibodies significantly reduced the residual disease.^186^ To test the effect and mechanisms of EGFR-TKI on esophageal squamous cell carcinoma (ESCC), Liu et al. treated ESCC PDX models with afatinib and examined the underlying mechanism. They discovered that afatinib exerts its function by suppressing EGFR downstream pathways and inducing cell apoptosis. Besides, aberrant phosphorylation of Src family kinases (SFKs) leads to resistance to afatinib, and the combination of EGFR and SFK inhibitors can overcome the afatinib resistance.^187^ Li et al. established three PDX models to explore the drug resistance mechanism of a pediatric BRAFV600E-mutant brain tumor against MEK inhibitor trametinib. They found that the decoupling of TORC1 signaling, originally a downstream pathway of MEK, led to resistance to trametinib; moreover, the combination of a TORC1 inhibitor and trametinib postponed the development of trametinib resistance.^188^

### Developing novel targeted drugs and exploring drug combinations

When utilizing targeted therapies to suppress a pathway that fuels cancer, a combination of drugs that aim at different “nodes” of the pathway would prevent bypass activation and promote cancer elimination. These combinations need preclinical validation to examine the efficacy and potential toxicity. As KRAS and BRAF mutations are identified as the drug-resistant mutations of cetuximab, Yao et al. established a PDX trial and validated that concurrent inhibition of RAS and EGFR had a synergistic effect on the treatment of BRAF and KRAS mutation cancer models.^48^ Zhao et al. established the HER2 + PDX model and confirmed the efficacy of neratinib, a pan-HER tyrosine kinase inhibitor, in combination with CDK4/6, mTOR, or MEK inhibitors in the context of HER2 + cancer.^189^ DNA-dependent protein kinase (DNA-PK) plays essential roles in DNA damage response and repair. Fok et al. combined a DNA-PK inhibitor, AZD7648, with chemotherapy or the PARP inhibitor olaparib to test its role as a potential sensitizer of other drugs targeting DNA repair. The synergistic effect on cell growth inhibition was validated, and the combination of AZD7648 and olaparib effectively increased the genomic instability.^190^ The IDH mutation has been an important target for acute myeloid leukemia (AML), which blocks AML cells’ differentiation. To increase the therapeutic effect of the IDH inhibitor, Liu et al. screened the genes that promote response to the IDH1 inhibitor, and they identified the critical role of STAT5 in stemness regulation. Correspondingly, combining an IDH1 inhibitor and a STAT5 inhibitor achieved a synergistic effect in IDH1 inhibitor-mutated AML PDX models.^191^

When cancer develops resistance to existing target therapies, the resistance mechanisms are distinct, and it is hard to find a one-size-fits-all compound that combats cancer. Furthermore, because of intratumor heterogeneity, the proportion of cells that develops a particular resistance mechanism is uncertain. In this scenario, PDX models satisfy the need to test the efficacy of new drug choices under corresponding genomic landscapes. HER-2 activating mutations led to resistance to cetuximab in colorectal cell lines. Kavuri et al. sequenced 48 colorectal cancer PDX models derived from patients resistant to cetuximab and discovered HER2 mutations in four models. The combination of two HER-2 targeted therapies produced tumor regression in the models.^19,192^ In gastrointestinal stromal tumors (GIST), tyrosine-protein kinase KIT has been a key target, while secondary KIT mutation is the central drug-resistance mechanism against tyrosine kinase inhibitors (TKI). Correspondingly, Gebreyohannes et al. tested avapritinib, a highly selective inhibitor of KIT, on GIST PDX models with different KIT mutations, which had a superior or equal therapeutic effect compared to standard TKI.^193^ Combining BRAF and MEK inhibitors has been an effective strategy for BRAF-mutant melanoma, and mTOR signaling is a pathway suppressed under this treatment. Therefore, when melanoma is resistant to this combination, one possible mechanism is an alternative activation of mTORC signaling. Therefore, Wang et al. established BRAF-mutant melanoma PDX models resistant to BRAF and MEK inhibitors and reduced their growth with mTOR inhibitors.^194^ Aberrant activation of STAT3 inhibitor has been a target for NSCLC because it contributes to the secondary resistance against EGFR-TKI. To deal with such resistance, Zheng et al. screened out a new STAT3 inhibitor, W2014-S, and proved its efficacy in NSCLC PDX models with abnormal STAT3 activation.^195^ Therefore, PDX models provide solid preclinical evidence of drug efficacy.

Moreover, regarding drugs designed for novel targets that have yet to be widely applied, preclinical results from PDX models provide solid evidence for further clinical trials. The Novartis Institutes for BioMedical Research established a PCT that followed the “one animal per model per treatment” design to simulate intertumor heterogeneity. In total, they tested the response to 62 treatment strategies.^13^ Aberrant activation of STAT3 inhibitor has been a target for NSCLC because it contributes to the secondary resistance against EGFR-TKI. To deal with such resistance, Zheng et al. screened out a new STAT3 inhibitor, W2014-S, and proved its efficacy in NSCLC PDX models with abnormal STAT3 activation. Moreover, W2014-S had a synergistic effect with the EGFR-TKI gefitinib on NSCLC PDX models that have acquired resistance to EGFR-TKIs.^195^ The IDH mutation has been an essential target for AML, which blocks AML cells’ differentiation. To increase the therapeutic effect of the IDH inhibitor, Liu et al. screened the genes that promoted response to the IDH1 inhibitor and identified STAT5 as a key node in stemness regulation. In IDH1 inhibitor-mutated AML PDX models, combining an IDH1 inhibitor and a STAT5 inhibitor achieved a synergistic effect.^191^

## PDX model in immunotherapy research

### Immune state and immunotherapy

Immunotherapy stands for a bright future for a wide variety of tumors. Its combination with other therapeutic regimens, including targeted therapy and many other forefront treatments yet to come, is a research hotspot that deserves full attention. However, the treatment efficacy lags,^196^ and only a limited cancer patient population benefits from immunotherapy. For instance, among CRC patients, only those with MSI status may benefit from immunotherapies, accounting only for 10% of all CRC patients.^197^ Cell-mediated immunity is essential for tumor immunity. Tumor cells present their antigens via the MHCI-antigen complex, and CD8 + T cells are activated when the CD8 molecule recognizes the tumor neoantigen by interacting directly with MHC-I.^198^ MSI results from the dysfunction of the mismatch repair system, which increases cancer neoantigen presentation, and MSI status thus indicates the patient’s response to immunotherapy.^199^ For the majority of tumor patients who are microsatellite stable (MSS), the cancer genome is less prone to mutate, and MSS cells are less likely to produce tumor antigens, which means that the immune response against cancer cells is not activated.^200,201^ Consequently, the MSS tumor immune microenvironment is “cold.” Researchers are exploring regimens to activate the immune response against cancer. Combining immunotherapy with targeted therapy, chemotherapy, radiotherapy, or alternative immunotherapies has improved the therapeutic effects. However, the immune status of patients cannot be fully captured, which makes it hard to keep track of the effects of the regimens. For example, it is impossible to take biopsies from patients after every stage of treatment. Therefore, models that simulate the immune landscape of cancer become necessary to improve existing therapies.

On the other hand, cytotoxic T cells are activated more easily for MSI patients, but intratumor heterogeneity still hinders the efficacy of immunotherapy. Mutations in different genetic loci in distinct cancer subclones may produce diversified tumor neoantigens, which are expressed dispersedly in relatively low levels. Consequently, these neoantigens cannot generate enough T-cell clones. Moreover, other factors, such as depletion of previous tumor antigens and dysfunction of MHC presentation, deprive the tumor-infiltrating lymphocytes of the original cytotoxic functions. However, traditional detection methods cannot fully capture the heterogeneity within a single patient. Similarly, an ‘avatar’ of the tumor in the human body is needed to dissect the overall situation of immune stimulation and improve the treatment against tumors.

### Exploring resistance mechanisms and combination therapy

Many unsolved problems in clinical practice seriously hamper the effectiveness of cancer treatment. PDX model as a feasible and reliable tool could and did help a lot in investigating the mechanisms of drug resistance and optimized combination therapies. In CRC PDX models, anti-PD-1 therapy strongly inhibited tumor growth in MSI models. In MSS models, it worked for the first ten days and was followed by a rapid tumor progression, which was consistent with clinical observation. The difference between the two groups was due to the disparate levels of infiltration and activation of immune cells in TME.^67^ These encouraging findings raise the question of what causes the immune barrier in MSS tumors and encourage us to dig deeper. Dysfunction and exhaustion of infiltrating immune cells in the TME are also hurdles to immunotherapy. A subset of CD8 + T cells was found to progressively expand in the TME, with the highest levels of proliferation and activation but the loss of IFN-γ production and the highest apoptotic tendency.^202^ This subset differed from the classic exhausted phenotype and was named the burned-out CD8 + T cell subset (Ebo). The abundance of Ebo was associated with immunotherapy resistance and could be minimized using avelumab (anti-PD-L1) in humanized PDX, indicating that the PD-1/PD-L1 pathway was crucial for its expansion, which was contradictory with prior studies revealing PD-1/PD-L1 as proliferation suppressors.^202–204^ The heterogeneity of cell subsets could be ubiquitous, and its impact on immunotherapy efficacy awaits further exploration.

PDX is also an excellent tool for testing the efficacy of potential combined therapies. Treatments that could turn the TME from immunosuppressive status into an immunoreactive status in a specific way would make the most of immunotherapy. IL-15 could promote NK cell maturation and function, with which the anti-disialoganglioside (GD2) antibody (mediating antibody-dependent cell-mediated cytotoxicity (ADCC)) exhibited an improved tumor-killing effect in neuroblastoma PDX.^205^ The immune response can be further activated by targeting CD105, specifically expressed on immunosuppressive cells such as mesenchymal stromal cells, tumor-associated macrophages, and cancer-associated fibroblasts. The relief of immunosuppression in the TME helped inhibit tumor growth more effectively in vivo.^206^ Studies found that thymocyte selection-associated high mobility group box protein (TOX) could bind to PD-1 in the cytoplasm and facilitate the endocytic recycling of PD1 in infiltrating CD8 + T cells, resulting in T cell exhaustion. TOX knockdown significantly alleviated exhaustion, increased TILs with upregulated IFN-γ and TNF-α, and maximized anti-PD-1 effectiveness in HCC PDX, proposing a new possibility of combination therapy.^207^ More interestingly, relieving tumor hypoxia by delivering exogenous H2O2 and catalase loaded within liposomes significantly reversed immunosuppressive TME and enhanced immunotherapy.^208^ Such a relatively simple and safe treatment opens a new direction for tumor therapy and is promising in translational medicine. Besides, many studies discovered a close relationship between the immune system and metabolism. Simvastatin, targeting cholesterol biosynthesis, inhibited lncRNA SNHG29-mediated YAP activation, thereby repressing PD-L1 expression at the transcriptional level. Knockdown of lncRNA SNHG29 promoted cytotoxic T lymphocyte (CTL) infiltration and killing in CRC PDX, suggesting a new mechanism for a classic drug.^209^ Other metabolism-related targets correlating with immune response, such as chondroitin-6-sulfate in CRC,^210^ could also be further validated in humanized PDX models.

### Optimizing CART therapy using PDX

How to specifically recognize tumor cells without off-tumor killing, augment infiltration of immune cells into tumor sites, and avert immune cell exhaustion induced by the immunosuppressive effect of the tumor microenvironment has long been a conundrum.^6^ Many savvy techniques have been applied in PDX models to enhance the performance of immune cells. As a classical target, HER-2 is highly expressed in tumors and lowly but extensively in normal tissues; hence, conventional HER-2 CART is potentially toxic to patients. Researchers designed an antibody-based switchable CAR T cell that bounded a specific peptide genetically engrafted onto a tumor-binding Fab molecule, the activity of which could be monitored in vivo based on dosage.^211^ As the target cells and immune cells connected, the “switch” took command of antigen specificity and T cell activation and induced complete tumor regression in PDX. Hunting for antigens with the highest tumor specificity is another way to optimize CART therapy. B7-H3 is a type I transmembrane protein highly expressed in human tumors and restrictedly expressed in normal tissues. It mediates the immunosuppressive process by reducing type I interferon released by T cells and cytotoxic activity by NK cells,^212,213^ making it an ideal immunotherapy target. With its antibody and antibody-drug conjugates being verified in PDX models,^214^ B7-H3.CAR-T was also tested in PDX and effectively controlled tumor growth without toxicity.^213^

Mining ways to extend the functional status of adoptive cells is also an important research direction. For instance, the combination of interleukin 17 (IL-17) and chemokine (C–C motif) ligand 19 (CCL19) could facilitate the formation and maintenance of T cell zones in the secondary lymphoid organs.^215,216^ Based on this, researchers constructed CAR T cells that simultaneously produced IL-17 and CCL19. These specific T cells led to an increase in many other tumor-infiltrating immune cells and the downregulation of immunosuppressive molecules such as PD-1 and TIGIT, exhibiting significant inhibition of tumor growth in PDX.^217^ Another design to treat GBM was a synthetic Notch receptor. It recognized either a tumor-specific but heterogeneous neoantigen (EGFRvIII) or a normal central nervous system-specific antigen (myelin oligodendrocyte glycoprotein). Subsequently, it activated a transcriptional output of a CAR that was designed against the more homogeneous GBM antigens, EphA2 and IL13Rα2.^218^ Such an approach complemented the antigens, increased the tumor-specificity, and enhanced CAR T cell persistence, providing ideas for improving CART treatment for other tumor types.^219^ In addition to PD-1 and PD-L1, which have been studied extensively in recent years, other immunoregulatory molecules with potent functions are being dug up, such as CD137, a costimulatory receptor on antigen-primed T cells, which could ameliorate immune cell exhaustion by reducing the expression of exhaustion-related genes.^220^ CARs designed to target CD137 significantly increased T cell proliferation and survival and upregulated IL-2, TNF-α, and IFN-γ in melanoma and GBM PDXs.^221–223^ Combining CD137 with known immunotherapy targets or tumor-specific antigens may open a new chapter in cancer treatment. In addition to longevity, the functioning of CART therapy is also plagued by alloreactivity caused by HLA-mismatched donated effector cells, which may lead to GvHD. Knockout of the endogenous TCRβ chain of CART cells significantly ablated alloreactivity and showed the same ability to control tumor burden in PDX models.^224^ However, it brought about low T-cell persistence compared to the co-expression of endogenous TCR plus CAR. Future studies may orchestrate these findings, and it is promising that we can have our cake and eat it too.

### Developing and testing novel forms and targets of immunotherapy

Existing immunotherapies are big steps forward in cancer treatment, but the beneficiaries are only a slight fraction, and the effective targets are still limited. It is thus imperative to devise new targets to complement the now available ones and develop new strategies to benefit patients more extensively. As a human body substitute, the PDX model has significant great contributions. The six-transmembrane epithelial antigen of the prostate (STEAP) is upregulated in multiple tumors and restricted in the normal tissue with T cell-engaging bispecific antibodies (T-BsAbs). Targeting STEAP in PDX effectively redirected T cells into tumor sites, thus significantly repressing tumor growth.^225^ An uncapped 5′-triphosphate moiety (ppp-RNA) of virus RNA could be recognized by the cytoplasmic immune receptor retinoic acid-inducible gene-I (RIG-I) and promote the production of type I interferon and pro-inflammatory cytokines.^226^ Based on this mechanism, ppp-RNA therapy was tested in PDX and demonstrated reduced tumor burden accompanied by CD3 + T cell expansion.^227^ CD47, which is expressed on both tumor and healthy cells, binds to its receptor signal-regulatory protein alpha (SIRPα) on myeloid cells to deliver a “do not eat me” signal.^228^ Preclinically, CAR T therapy targeting highly expressed CD33 in acute myeloid leukemia is effective with no obvious toxicity in both in vitro and in vivo PDX models, allowing it to enter the first-ever human phase 1 clinical trial, completing the leap from bench to bed.^229^ CD47 blockade could fire innate immunity by triggering phagocytosis. In PDX models, it reduces liver micrometastasis and prolongs survival,^230,231^ offering a solid complement for clinical immunotherapy practice. Ex vivo expansion of NK cells from cancer patients possessed excellent tumor-killing ability in PDX under the administration of IL-2 injection to maintain cell viability, providing a promising path to reconstitute the patients’ exhausted immune function compatible with existing immunotherapies.^232^

## PDX model in novel therapies

Antibody-drug conjugates (ADCs) are a novel therapy that utilizes antibodies to target specific oncogenes and delivers the drugs to kill the cancer cells specifically. PDX models have been extensively used in the preclinical experiments of ADCs. A critical mechanism for patients resistant to EFGR inhibitors is the overexpression of HER3, which forms dimers with EGFR and HER2 as an alternative pathway. Haikala et al. treated EGFR-resistant PDX models effectively with HER3-Dxd, an ADC conjugated with the HER3 antibody. Moreover, when pretreated with an EGFR-TKI osimertinib, the membrane expression of HER3 was increased, and the efficacy of HER3-Dxd improved.^233^ Another anti-HER3 antibody-drug conjugate, EV20/MMAF, demonstrated potent anti-tumoral properties in several primary and secondary resistance models to commonly available anti-HER2 therapies.^234^

Besides, PDX is considered a persuasive tool to validate the efficacy of a new drug in the preclinical stage.^235^ PDX models have been extensively used to assess the efficacy of new potential drugs or carriers, such as microRNA,^236^ non-coding RNA,^237^ small-molecule inhibitors,^238^ and novel drug carriers such as functionalized hyaluronic acid^239^ and quantum dot.^240^ Moreover, PDX models can be used in designing radionuclide therapy, as they can recapitulate the biodistribution of radiotracers.^241^ The potential drugs that were validated in PDX models have been summarized in Table 5.

**Table 5:** PDX model in exploring novel therapy

Name	Category	PDX origin	Mechanism
miR-145^236^	Non-coding RNA	CRC	Reversing snail family transcriptional repressor 1 (SNAI1)-mediated stemness and radiation resistance (RT)
CircPTK2^237^	Non-coding RNA	CRC	Promoting EMT of CRC cells in vitro and in vivo by binding to vimentin protein on sites Ser38, Ser55 and Ser82
MRTX849^286^	Small-molecule inhibitors	Colon cancer	Inhibiting KRAS G12C in CRC
Silvestrol^238^	Small-molecule inhibitors	CRC	Inhibiting EIF4A2 and inhibiting CRC invasion and migration, sphere formation and enhanced sensitivity to oxaliplatin treatment
S63845^287^	Small-molecule inhibitors	CRC	Inhibiting Mcl-1 and re-sensitizing CRC with intrinsic and acquired regorafenib resistance in vitro and in vivo
PTK6^288^	Small-molecule inhibitors	CRC	Promoting the stemness of CRC cells through interacting with JAK2 and phosphorylating it to activate the JAK2/STAT3 signaling.
BET inhibitors^289^	Small-molecule inhibitors	Colon cancer	BETi combined with chemotherapy suppressed the tumor growth in a DR5-dependent manner and potently inhibited patient-derived xenograft tumor growth with enhanced DR5 induction and apoptosis.
MDL-811.^290^	Small-molecule activator	CRC	Activating SIRT6 histone H3 deacetylation (H3K9Ac, H3K18Ac, and H3K56Ac) in vitro and had broad antiproliferative effects on diverse CRC cell lines and PDOs.
RGX-202^291^	Small-molecule activator	CRC	Suppressing CRC growth across KRAS wild-type and KRAS mutant xenograft, syngeneic, and patient-derived xenograft (PDX) tumors.
ONC201^292^	Small-molecule activator	CRC	TRAIL enhancer ONC201 combined with anti-angiogenic therapies such as bevacizumab increasing tumor cell death and inhibiting proliferation.
Dehydrodiisoeugenol^293^	Natural extracts	CRC	Inducing strong cellular autophagy, which could be inhibited through autophagic inhibitors, with activation in the DEH-induced inhibition of cell growth in CRC
Asparaginase^294^	Amino acid enzyme	CRC	Toxic to CRC with WNT-activating mutations that inhibit GSK3
Autologous extracellular vesicles^295^	Extracellular vesicles	CRC	Transplanted EVs recognizing tumors from the cognate nanoparticle-generating individual, suggesting the theranostic potential of autologous EVs.
TCP1-CD-QD nanocarriers^240^	Quantum dot nanocarriers	CRC	5-FU and miR-34a(m) can be efficiently encapsulated into TCP1-CD-QD nanocarriers and delivered into CRC cells, which led to the inhibition of the proliferation and migration of CRC cells
SNP^296^	Nanometer materials	Colon cancer	Spatiotemporally delivering CA4P to tumor neovasculature and bortezomib to tumor cells mediated by the site-specific stimuli-activated drug release.
Human serum albumin nanoparticles (HSA NPs) containing 5-FU^297^	Nanometer materials	CRC	Accurately targeting the tumor designated by LRP-1 responding radiation and showing therapeutic efficacy in the radio-resistant PDX model.
Π electron-stabilized polymeric micelles^298^	Nanometer materials	GI cancers	Loading docetaxel and potentiating chemotherapy responses in multiple advanced-stage GI cancer mouse models.
Peptide functionalized hyaluronic acid^239^	Bioavailable peptide	glioblastoma	Treatment with drug-loaded hydrogels allowed for a significant survival impact of 45%

## Conclusion

Biomarker-based therapies, including targeted therapy and immunotherapy, have been the research focus in recent years to extend patients’ survival times, and encouragingly, they have shown promising therapeutic efficacy for a certain number of patients. However, how to recognize and stratify patients with different response potencies accurately using current and untapped biomarkers is still a conundrum. Current biomarkers are insufficient to mirror the genuine genetic status due to the ubiquitous inter- and intra-tumor heterogeneity, which is regarded as the “villain” in drug resistance. Even if novel biomarkers or signatures accurately reflect the cancer landscape, the consequent problem is the complexity of treatment selection. There are uncertain interactions between these biomarkers and inconclusive therapeutic effects on different patients. It is hence vital to perform expanded validation of treatment-related decision-making in reliable preclinical models.

PDX is currently the best model to retain the original features of the tumor. More importantly, it became possible to mimic the interactions between the tumor and the immune system in vivo by establishing humanized PDX. It is known that there is a mutual influence between the tumor and the immune system, and they both adapt and make changes during their interactions. The emergence of humanized PDX makes for an optimized model and a more suitable platform for studying immunotherapy. Because PDX could preserve the genetic landscape of the parental tumor and parallel the clonal heterogeneity to a large extent, therapeutic effect testing and drug resistance exploration in PDX could reflect the real situation of cancer to the utmost. Intricate associations between classical biomarkers such as HER2, KRAS, and BRAF and drug response have been broadly investigated in PDX to find more clues to improve drug efficiency. Combination therapy and newly developed drugs could also be screened and validated in PDX to get reliable preclinical data for translational research. In addition, the availability of PDX allows for longitudinal observation of tumor heterogeneity and some characteristics, such as clonal metastasis tendency. PDX could be used to achieve personalized patient medication management, which is the ultimate translational goal.

In addition to the advantages mentioned above, PDX still has some deficiencies to be solved. To perfect PDX models, efforts like improving transplantation methods and genetic modification have been made, coming at the cost of time and money. Moreover, novel support technologies such as multi-omics, massive data, and functional genomics facilitate the analysis of the PDX model’s properties. All these considerations highlight the promising futures offered by PDX to light the way for translational cancer research. However, they also put forward the inherent challenges to this method and the need to mine new ways to maximize the potential of PDX.

## References

[1] Letai A, Bhola P, Welm AL (2022). Functional precision oncology: testing tumors with drugs to identify vulnerabilities and novel combinations. Cancer Cell.

[2] Woolston A (2019). Genomic and transcriptomic determinants of therapy resistance and immune landscape evolution during anti-EGFR treatment in colorectal cancer. Cancer Cell.

[3] Chen K (2019). Perioperative dynamic changes in circulating tumor DNA in patients with lung cancer (DYNAMIC). Clin. Cancer Res..

[4] Hay M (2014). Clinical development success rates for investigational drugs. Nat. Biotechnol..

[5] Marusyk A, Janiszewska M, Polyak K (2020). Intratumor heterogeneity: the rosetta stone of therapy resistance. Cancer Cell.

[6] Wu W (2021). Intratumor heterogeneity: the hidden barrier to immunotherapy against MSI tumors from the perspective of IFN-gamma signaling and tumor-infiltrating lymphocytes. J. Hematol. Oncol..

[7] Ramzy GM (2020). Patient-derived in vitro models for drug discovery in colorectal carcinoma. Cancers.

[8] Johnson JI (2001). Relationships between drug activity in NCI preclinical in vitro and in vivo models and early clinical trials. Br. J. Cancer.

[9] Dsouza VL, Kuthethur R, Kabekkodu SP, Chakrabarty S (2022). Organ-on-Chip platforms to study tumor evolution and chemosensitivity. Biochim. Biophys. Acta Rev. Cancer.

[10] Carvalho MR (2019). Colorectal tumor-on-a-chip system: a 3D tool for precision onco-nanomedicine. Sci. Adv..

[11] Xu R, Zhou X, Wang S, Trinkle C (2021). Tumor organoid models in precision medicine and investigating cancer-stromal interactions. Pharmacol. Ther..

[12] Hidalgo M (2014). Patient-derived xenograft models: an emerging platform for translational cancer research. Cancer Discov..

[13] Gao H (2015). High-throughput screening using patient-derived tumor xenografts to predict clinical trial drug response. Nat. Med..

[14] Rygaard J, Povlsen CO (1969). Heterotransplantation of a human malignant tumour to “Nude” mice. Acta Pathol. Microbiol. Scand..

[15] Fiebig HH (1984). Comparison of tumor response in nude mice and in the patients. Behring Inst. Mitt..

[16] Hutchinson L, Kirk R (2011). High drug attrition rates-where are we going wrong?. Nat. Rev. Clin. Oncol..

[17] Fichtner I (2004). Anticancer drug response and expression of molecular markers in early-passage xenotransplanted colon carcinomas. Eur. J. Cancer.

[18] Rubio-Viqueira B (2006). An in vivo platform for translational drug development in pancreatic cancer. Clin. Cancer Res..

[19] Bertotti A (2011). A molecularly annotated platform of patient-derived xenografts (“xenopatients”) identifies HER2 as an effective therapeutic target in cetuximab-resistant colorectal cancer. Cancer Discov..

[20] Bock BC, Stein U, Schmitt CA, Augustin HG (2014). Mouse models of human cancer. Cancer Res..

[21] Abdolahi S (2022). Patient-derived xenograft (PDX) models, applications and challenges in cancer research. J. Transl. Med..

[22] Okada S, Vaeteewoottacharn K, Kariya R (2019). Application of Highly Immunocompromised Mice for the Establishment of Patient-Derived Xenograft (PDX) Models. Cells.

[23] Invrea F (2020). Patient-derived xenografts (PDXs) as model systems for human cancer. Curr. Opin. Biotechnol..

[24] Collins AT, Lang SH (2018). A systematic review of the validity of patient derived xenograft (PDX) models: the implications for translational research and personalised medicine. PeerJ.

[25] Katsiampoura A (2017). Modeling of patient-derived xenografts in colorectal cancer. Mol. Cancer Ther..

[26] Byrne AT (2017). Interrogating open issues in cancer precision medicine with patient-derived xenografts. Nat. Rev. Cancer.

[27] Martins-Filho SN (2020). EGFR-mutated lung adenocarcinomas from patients who progressed on EGFR-inhibitors show high engraftment rates in xenograft models. Lung Cancer.

[28] Jung HY (2020). PDX models of human lung squamous cell carcinoma: consideration of factors in preclinical and co-clinical applications. J. Transl. Med..

[29] Zhu M (2020). Uncovering biological factors that regulate hepatocellular carcinoma growth using patient-derived xenograft assays. Hepatology.

[30] Richter A (2022). The molecular subtype of adult acute lymphoblastic leukemia samples determines the engraftment site and proliferation kinetics in patient-derived xenograft models. Cells.

[31] Yu J (2017). Establishing and characterizing patient-derived xenografts using pre-chemotherapy percutaneous biopsy and post-chemotherapy surgical samples from a prospective neoadjuvant breast cancer study. Breast Cancer Res..

[32] Boughey JC (2021). Patient-derived xenograft engraftment and breast cancer outcomes in a prospective neoadjuvant study (BEAUTY). Clin. Cancer Res..

[33] Nemati F (2010). Establishment and characterization of a panel of human uveal melanoma xenografts derived from primary and/or metastatic tumors. Clin. Cancer Res..

[34] Saito Y, Shultz LD, Ishikawa F (2020). Understanding normal and malignant human hematopoiesis using next-generation humanized mice. Trends Immunol..

[35] Shultz LD, Ishikawa F, Greiner DL (2007). Humanized mice in translational biomedical research. Nat. Rev. Immunol..

[36] Ito M (2002). NOD/SCID/gamma(c)(null) mouse: an excellent recipient mouse model for engraftment of human cells. Blood.

[37] Ishikawa F (2005). Development of functional human blood and immune systems in NOD/SCID/IL2 receptor gamma chain(null) mice. Blood.

[38] Melkus MW (2006). Humanized mice mount specific adaptive and innate immune responses to EBV and TSST-1. Nat. Med..

[39] Tary-Lehmann M (1994). Anti-SCID mouse reactivity shapes the human CD4+ T cell repertoire in hu-PBL-SCID chimeras. J. Exp. Med..

[40] van Rijn RS (2003). A new xenograft model for graft-versus-host disease by intravenous transfer of human peripheral blood mononuclear cells in RAG2-/- gammac-/- double-mutant mice. Blood.

[41] Meraz IM (2019). An improved patient-derived xenograft humanized mouse model for evaluation of lung cancer immune responses. Cancer Immunol. Res..

[42] Horowitz NB (2021). Humanized mouse models for the advancement of innate lymphoid cell-based cancer immunotherapies. Front. Immunol..

[43] Bareham B (2021). Modeling human tumor-immune environments in vivo for the preclinical assessment of immunotherapies. Cancer Immunol. Immunother..

[44] Izumchenko E (2017). Patient-derived xenografts effectively capture responses to oncology therapy in a heterogeneous cohort of patients with solid tumors. Ann. Oncol..

[45] Guenot D (2006). Primary tumour genetic alterations and intra-tumoral heterogeneity are maintained in xenografts of human colon cancers showing chromosome instability. J. Pathol..

[46] Wang J (2019). Molecularly annotation of mouse avatar models derived from patients with colorectal cancer liver metastasis. Theranostics.

[47] Vaubel RA (2020). Genomic and phenotypic characterization of a broad panel of patient-derived xenografts reflects the diversity of glioblastoma. Clin. Cancer Res..

[48] Yao YM (2017). Mouse PDX trial suggests synergy of concurrent inhibition of RAF and EGFR in colorectal cancer with BRAF or KRAS mutations. Clin. Cancer Res..

[49] Zhao Y (2021). Diverse alterations associated with resistance to KRAS(G12C) inhibition. Nature.

[50] Georgopoulou D (2021). Landscapes of cellular phenotypic diversity in breast cancer xenografts and their impact on drug response. Nat. Commun..

[51] Sveen A (2018). Colorectal cancer consensus molecular subtypes translated to preclinical models uncover potentially targetable cancer cell dependencies. Clin. Cancer Res..

[52] Julien S (2012). Characterization of a large panel of patient-derived tumor xenografts representing the clinical heterogeneity of human colorectal cancer. Clin. Cancer Res..

[53] Liu J (2019). Pathological pattern of intrahepatic HBV in HCC is phenocopied by PDX-derived mice: a novel model for antiviral treatment. Transl. Oncol..

[54] Braekeveldt N (2018). Patient-derived xenograft models reveal intratumor heterogeneity and temporal stability in neuroblastoma. Cancer Res..

[55] Dahlmann M (2021). Peritoneal metastasis of colorectal cancer (pmCRC): identification of predictive molecular signatures by a novel preclinical platform of matching pmCRC PDX/PD3D models. Mol. Cancer.

[56] Cho SY (2019). Unstable genome and transcriptome dynamics during tumor metastasis contribute to therapeutic heterogeneity in colorectal cancers. Clin. Cancer Res..

[57] Zhang Z (2020). A novel patient-derived orthotopic xenograft (PDOX) mouse model of highly-aggressive liver metastasis for identification of candidate effective drug-combinations. Sci. Rep..

[58] Want MY (2019). Neoantigens retention in patient derived xenograft models mediates autologous T cells activation in ovarian cancer. Oncoimmunology.

[59] Rijensky NM (2020). Identification of tumor antigens in the HLA peptidome of patient-derived xenograft tumors in mouse. Mol. Cell. Proteom..

[60] Liu WN (2020). Establishment and characterization of humanized mouse NPC-PDX model for testing immunotherapy. Cancers.

[61] Shin JH (2021). Colon cancer cells acquire immune regulatory molecules from tumor-infiltrating lymphocytes by trogocytosis. Proc. Natl Acad. Sci. USA.

[62] Zhao Y (2018). Development of a new patient-derived xenograft humanised mouse model to study human-specific tumour microenvironment and immunotherapy. Gut.

[63] Le DT (2021). Natural killer cells and cytotoxic T lymphocytes are required to clear solid tumor in a patient-derived xenograft. JCI Insight.

[64] Brehm MA (2019). Lack of acute xenogeneic graft- versus-host disease, but retention of T-cell function following engraftment of human peripheral blood mononuclear cells in NSG mice deficient in MHC class I and II expression. FASEB J..

[65] Covassin L (2013). Human immune system development and survival of non-obese diabetic (NOD)-scid IL2rgamma(null) (NSG) mice engrafted with human thymus and autologous haematopoietic stem cells. Clin. Exp. Immunol..

[66] King MA (2009). Human peripheral blood leucocyte non-obese diabetic-severe combined immunodeficiency interleukin-2 receptor gamma chain gene mouse model of xenogeneic graft-versus-host-like disease and the role of host major histocompatibility complex. Clin. Exp. Immunol..

[67] Capasso A (2019). Characterization of immune responses to anti-PD-1 mono and combination immunotherapy in hematopoietic humanized mice implanted with tumor xenografts. J. Immunother. Cancer.

[68] Bleijs M, van de Wetering M, Clevers H, Drost J (2019). Xenograft and organoid model systems in cancer research. EMBO J..

[69] Rizzo G, Bertotti A, Leto SM, Vetrano S (2021). Patient-derived tumor models: a more suitable tool for pre-clinical studies in colorectal cancer. J. Exp. Clin. Cancer Res..

[70] Nunes M (2015). Evaluating patient-derived colorectal cancer xenografts as preclinical models by comparison with patient clinical data. Cancer Res..

[71] Yoshida GJ (2020). Applications of patient-derived tumor xenograft models and tumor organoids. J. Hematol. Oncol..

[72] Guo W, Giancotti FG (2004). Integrin signalling during tumour progression. Nat. Rev. Mol. Cell Biol..

[73] Blomme A (2018). Murine stroma adopts a human-like metabolic phenotype in the PDX model of colorectal cancer and liver metastases. Oncogene.

[74] Collins MK (1989). Species specificity of interleukin 2 binding to individual receptor components. Eur. J. Immunol..

[75] Huntington ND (2009). IL-15 trans-presentation promotes human NK cell development and differentiation in vivo. J. Exp. Med..

[76] Siolas D, Hannon GJ (2013). Patient-derived tumor xenografts: transforming clinical samples into mouse models. Cancer Res..

[77] Hoogstad-van Evert JS (2017). Umbilical cord blood CD34(+) progenitor-derived NK cells efficiently kill ovarian cancer spheroids and intraperitoneal tumors in NOD/SCID/IL2Rg(null) mice. Oncoimmunology.

[78] Rongvaux A (2014). Development and function of human innate immune cells in a humanized mouse model. Nat. Biotechnol..

[79] Zeleniak A (2022). De novo construction of T cell compartment in humanized mice engrafted with iPSC-derived thymus organoids. Nat. Methods.

[80] Guinney J (2015). The consensus molecular subtypes of colorectal cancer. Nat. Med..

[81] Isella C (2015). Stromal contribution to the colorectal cancer transcriptome. Nat. Genet..

[82] Isella C (2017). Selective analysis of cancer-cell intrinsic transcriptional traits defines novel clinically relevant subtypes of colorectal cancer. Nat. Commun..

[83] Dang, H. X. et al. The clonal evolution of metastatic colorectal cancer. *Sci. Adv*. **6**, eaay9691 (2020). 10.1126/sciadv.aay9691 PMC7286679 32577507

[84] Kerstetter-Fogle AE, Harris PLR, Brady-Kalnay SM, Sloan AE (2020). Generation of glioblastoma patient-derived intracranial xenografts for preclinical studies. Int. J. Mol. Sci..

[85] Hoge ACH (2022). DNA-based copy number analysis confirms genomic evolution of PDX models. NPJ Precis. Oncol..

[86] Prasetyanti PR (2019). Capturing colorectal cancer inter-tumor heterogeneity in patient-derived xenograft (PDX) models. Int. J. Cancer.

[87] Linnekamp JF (2018). Consensus molecular subtypes of colorectal cancer are recapitulated in in vitro and in vivo models. Cell Death Differ..

[88] Suto H (2021). Microsatellite instability-high colorectal cancer patient-derived xenograft models for cancer immunity research. J. Cancer Res. Ther..

[89] Liggett PE (1993). Heterotransplantation of human uveal melanoma. Graefes Arch. Clin. Exp. Ophthalmol..

[90] Lissa D (2022). Heterogeneity of neuroendocrine transcriptional states in metastatic small cell lung cancers and patient-derived models. Nat. Commun..

[91] Chateau-Joubert S (2021). Spontaneous mouse lymphoma in patient-derived tumor xenografts: The importance of systematic analysis of xenografted human tumor tissues in preclinical efficacy trials. Transl. Oncol..

[92] Dieter SM (2017). Patient-derived xenografts of gastrointestinal cancers are susceptible to rapid and delayed B-lymphoproliferation. Int. J. Cancer.

[93] Butler KA (2017). Prevention of human lymphoproliferative tumor formation in ovarian cancer patient-derived xenografts. Neoplasia.

[94] Zhang F (2018). Characterization of drug responses of mini patient-derived xenografts in mice for predicting cancer patient clinical therapeutic response. Cancer Commun..

[95] Liu Z (2020). A fast, simple, and cost-effective method of expanding patient-derived xenograft mouse models of pancreatic ductal adenocarcinoma. J. Transl. Med..

[96] Lazzari L (2019). Patient-derived xenografts and matched cell lines identify pharmacogenomic vulnerabilities in colorectal cancer. Clin. Cancer Res..

[97] Luraghi P (2018). A molecularly annotated model of patient-derived colon cancer stem-like cells to assess genetic and nongenetic mechanisms of resistance to anti-EGFR therapy. Clin. Cancer Res..

[98] Guillen KP (2022). A human breast cancer-derived xenograft and organoid platform for drug discovery and precision oncology. Nat. Cancer.

[99] Huang L (2020). PDX-derived organoids model in vivo drug response and secrete biomarkers. JCI Insight.

[100] Hassani, I. et al. Engineered colorectal cancer tissue recapitulates key attributes of a patient-derived xenograft tumor line. *Biofabrication*. **14**, 10.1088/1758-5090/ac73b6 (2022). 10.1088/1758-5090/ac73b6 PMC9822569 35617932

[101] Amaral R (2020). A Simple Three-Dimensional In Vitro Culture Mimicking the In Vivo-Like Cell Behavior of Bladder Patient-Derived Xenograft Models. Cancers (Basel)..

[102] Ice RJ (2020). Drug responses are conserved across patient-derived xenograft models of melanoma leading to identification of novel drug combination therapies. Br. J. Cancer.

[103] Xiao J, Glasgow E, Agarwal S (2020). Zebrafish xenografts for drug discovery and personalized medicine. Trends Cancer.

[104] Almstedt E (2022). Real-time evaluation of glioblastoma growth in patient-specific zebrafish xenografts. Neuro Oncol..

[105] Ali Z (2022). Zebrafish patient-derived xenograft models predict lymph node involvement and treatment outcome in non-small cell lung cancer. J. Exp. Clin. Cancer Res..

[106] Pizon M (2022). Chick chorioallantoic membrane (CAM) assays as a model of patient-derived xenografts from circulating cancer stem cells (cCSCs) in breast cancer patients. Cancers.

[107] Sun H (2021). Comprehensive characterization of 536 patient-derived xenograft models prioritizes candidatesfor targeted treatment. Nat. Commun..

[108] Dudova Z (2022). The EurOPDX Data Portal: an open platform for patient-derived cancer xenograft data sharing and visualization. BMC Genomics.

[109] Evrard YA (2020). Systematic establishment of robustness and standards in patient-derived xenograft experiments and analysis. Cancer Res..

[110] Meehan TF (2017). PDX-MI: minimal information for patient-derived tumor xenograft models. Cancer Res..

[111] Woo XY (2022). A genomically and clinically annotated patient-derived xenograft resource for preclinical research in non-small cell lung cancer. Cancer Res..

[112] Mullins, C. S. et al. Integrated biobanking and tumor model establishment of human colorectal carcinoma provides excellent tools for preclinical research. *Cancers*. **11** (2019). 10.3390/cancers11101520 PMC6826890 31601052

[113] Corso S (2019). A comprehensive PDX gastric cancer collection captures cancer cell-intrinsic transcriptional MSI traits. Cancer Res..

[114] Conte N (2019). PDX Finder: a portal for patient-derived tumor xenograft model discovery. Nucleic Acids Res..

[115] Yaegashi M (2021). Frequent post-operative monitoring of colorectal cancer using individualised ctDNA validated by multiregional molecular profiling. Br. J. Cancer.

[116] Cayrefourcq L (2021). Selective treatment pressure in colon cancer drives the molecular profile of resistant circulating tumor cell clones. Mol. Cancer.

[117] Russo M (2016). Acquired resistance to the TRK inhibitor entrectinib in colorectal cancer. Cancer Discov..

[118] Eslami SZ (2022). Functional analysis of circulating tumour cells: the KEY to understand the biology of the metastatic cascade. Br. J. Cancer.

[119] Faugeroux V (2020). Genetic characterization of a unique neuroendocrine transdifferentiation prostate circulating tumor cell-derived eXplant model. Nat. Commun..

[120] Wei X (2020). Targeted CRISPR screening identifies PRMT5 as synthetic lethality combinatorial target with gemcitabine in pancreatic cancer cells. Proc. Natl Acad. Sci. USA.

[121] Bossi D (2016). In vivo genetic screens of patient-derived tumors revealed unexpected frailty of the transformed phenotype. Cancer Discov..

[122] Lin S (2022). An in vivo CRISPR screening platform for prioritizing therapeutic targets in AML. Cancer Discov..

[123] Hulton CH (2020). Direct genome editing of patient-derived xenografts using CRISPR-Cas9 enables rapid in vivo functional genomics. Nat. Cancer.

[124] Wirth AK (2022). In vivo PDX CRISPR/Cas9 screens reveal mutual therapeutic targets to overcome heterogeneous acquired chemo-resistance. Leukemia.

[125] Carlet M (2021). In vivo inducible reverse genetics in patients’ tumors to identify individual therapeutic targets. Nat. Commun..

[126] Liu Y (2020). High-spatial-resolution multi-omics sequencing via deterministic barcoding in tissue. Cell.

[127] Yaari Z (2016). Theranostic barcoded nanoparticles for personalized cancer medicine. Nat. Commun..

[128] Roche S (2020). Establishment and characterisation by expression microarray of patient-derived xenograft panel of human pancreatic adenocarcinoma patients. Int. J. Mol. Sci..

[129] Sueyoshi K (2021). Multi-tumor analysis of cancer-stroma interactomes of patient-derived xenografts unveils the unique homeostatic process in renal cell carcinomas. iScience.

[130] Mirhadi S (2022). Integrative analysis of non-small cell lung cancer patient-derived xenografts identifies distinct proteotypes associated with patient outcomes. Nat. Commun..

[131] Kaoutari AE (2021). Metabolomic profiling of pancreatic adenocarcinoma reveals key features driving clinical outcome and drug resistance. EBioMedicine.

[132] Yao Y (2022). Clinical utility of PDX cohorts to reveal biomarkers of intrinsic resistance and clonal architecture changes underlying acquired resistance to cetuximab in HNSCC. Signal Transduct. Target Ther..

[133] Dimitrov-Markov S (2020). Discovery of new targets to control metastasis in pancreatic cancer by single-cell transcriptomics analysis of circulating tumor cells. Mol. Cancer Ther..

[134] Mori H (2021). Influence of estrogen treatment on ESR1(+) and ESR1(-) cells in ER(+) breast cancer: insights from single-cell analysis of patient-derived xenograft models. Cancers.

[135] Grosselin K (2019). High-throughput single-cell ChIP-seq identifies heterogeneity of chromatin states in breast cancer. Nat. Genet..

[136] Sato K (2019). Multiregion genomic analysis of serially transplanted patient-derived xenograft tumors. Cancer Genomics Proteom..

[137] Cheng X (2021). SPA: a quantitation strategy for MS data in patient-derived xenograft models. Genomics Proteom. Bioinforma..

[138] Tomar T (2016). Genome-wide methylation profiling of ovarian cancer patient-derived xenografts treated with the demethylating agent decitabine identifies novel epigenetically regulated genes and pathways. Genome Med..

[139] Lenkiewicz E (2020). Genomic and epigenomic landscaping defines new therapeutic targets for adenosquamous carcinoma of the pancreas. Cancer Res..

[140] Tedesco M (2022). Chromatin Velocity reveals epigenetic dynamics by single-cell profiling of heterochromatin and euchromatin. Nat. Biotechnol..

[141] La Manno G (2018). RNA velocity of single cells. Nature.

[142] Franciosa G (2021). Proteomics of resistance to Notch1 inhibition in acute lymphoblastic leukemia reveals targetable kinase signatures. Nat. Commun..

[143] Contreras-Trujillo H (2021). Deciphering intratumoral heterogeneity using integrated clonal tracking and single-cell transcriptome analyses. Nat. Commun..

[144] Peng D (2021). Evaluating the transcriptional fidelity of cancer models. Genome Med..

[145] Li Q (2019). DRAP: a toolbox for drug response analysis and visualization tailored for preclinical drug testing on patient-derived xenograft models. J. Transl. Med..

[146] Kim Y (2020). PDXGEM: patient-derived tumor xenograft-based gene expression model for predicting clinical response to anticancer therapy in cancer patients. BMC Bioinform..

[147] Mer AS (2019). Integrative pharmacogenomics analysis of patient-derived xenografts. Cancer Res..

[148] Mourragui S (2019). PRECISE: a domain adaptation approach to transfer predictors of drug response from pre-clinical models to tumors. Bioinformatics.

[149] Mourragui SMC (2021). Predicting patient response with models trained on cell lines and patient-derived xenografts by nonlinear transfer learning. Proc. Natl Acad. Sci. USA.

[150] Ma J (2021). Few-shot learning creates predictive models of drug response that translate from high-throughput screens to individual patients. Nat. Cancer.

[151] Tiwari V (2020). In vivo MRS measurement of 2-hydroxyglutarate in patient-derived IDH-mutant xenograft mouse models versus glioma patients. Magn. Reson. Med..

[152] Jardim-Perassi BV (2021). Deep-learning and MR images to target hypoxic habitats with evofosfamide in preclinical models of sarcoma. Theranostics.

[153] Roy S (2020). Optimal co-clinical radiomics: sensitivity of radiomic features to tumour volume, image noise and resolution in co-clinical T1-weighted and T2-weighted magnetic resonance imaging. EBioMedicine.

[154] Roy S (2022). Co-clinical FDG-PET radiomic signature in predicting response to neoadjuvant chemotherapy in triple-negative breast cancer. Eur. J. Nucl. Med Mol. Imaging.

[155] Moss JI (2020). High-resolution 3D visualization of nanomedicine distribution in tumors. Theranostics.

[156] Russell J (2021). Predicting gemcitabine delivery by (18)F-FAC PET in murine models of pancreatic cancer. J. Nucl. Med..

[157] Kleinmanns K (2020). CD24-targeted intraoperative fluorescence image-guided surgery leads to improved cytoreduction of ovarian cancer in a preclinical orthotopic surgical model. EBioMedicine.

[158] Kleinmanns K (2020). CD24-targeted fluorescence imaging in patient-derived xenograft models of high-grade serous ovarian carcinoma. EBioMedicine.

[159] Fonnes T (2020). Near-infrared fluorescent imaging for monitoring of treatment response in endometrial carcinoma patient-derived xenograft models. Cancers (Basel)..

[160] Luo D (2019). Pharmacokinetics and pharmacodynamics of liposomal chemophototherapy with short drug-light intervals. J. Control Release.

[161] Honkala A, Malhotra SV, Kummar S, Junttila MR (2022). Harnessing the predictive power of preclinical models for oncology drug development. Nat. Rev. Drug Discov..

[162] Zhan M (2018). Guided chemotherapy based on patient-derived mini-xenograft models improves survival of gallbladder carcinoma patients. Cancer Commun..

[163] Loftus JP (2021). Combinatorial efficacy of entospletinib and chemotherapy in patient-derived xenograft models of infant acute lymphoblastic leukemia. Haematologica.

[164] Li LY (2019). Genetic Profiles associated with chemoresistance in patient-derived xenograft models of ovarian cancer. Cancer Res. Treat..

[165] Yue J (2020). Targeted chemotherapy overcomes drug resistance in melanoma. Genes Dev..

[166] Dilly AK (2021). Improved chemosensitivity following mucolytic therapy in patient-derived models of mucinous appendix cancer. Transl. Res..

[167] Inkoom A (2020). Enhancing efficacy of gemcitabine in pancreatic patient-derived xenograft mouse models. Int. J. Pharm. X.

[168] Tan P (2021). Enhanced chemo-photodynamic therapy of an enzyme-responsive prodrug in bladder cancer patient-derived xenograft models. Biomaterials.

[169] Tanaka N (2021). Clinical acquired resistance to KRAS(G12C) inhibition through a novel KRAS switch-II pocket mutation and polyclonal alterations converging on RAS-MAPK reactivation. Cancer Discov..

[170] Russo M (2019). Adaptive mutability of colorectal cancers in response to targeted therapies. Science.

[171] Amodio V (2020). EGFR blockade reverts resistance to KRAS(G12C) inhibition in colorectal cancer. Cancer Discov..

[172] Chen Z (2018). Characterization and validation of potential therapeutic targets based on the molecular signature of patient-derived xenografts in gastric cancer. J. Hematol. Oncol..

[173] Schutte M (2017). Molecular dissection of colorectal cancer in pre-clinical models identifies biomarkers predicting sensitivity to EGFR inhibitors. Nat. Commun..

[174] Lindner AU (2020). Systems analysis of protein signatures predicting cetuximab responses in KRAS, NRAS, BRAF and PIK3CA wild-type patient-derived xenograft models of metastatic colorectal cancer. Int. J. Cancer.

[175] Bonazzi VF (2022). Patient-derived xenograft models capture genomic heterogeneity in endometrial cancer. Genome Med..

[176] Fukamachi H (2019). A subset of diffuse-type gastric cancer is susceptible to mTOR inhibitors and checkpoint inhibitors. J. Exp. Clin. Cancer Res..

[177] Kemper K (2016). BRAF(V600E) kinase domain duplication identified in therapy-refractory melanoma patient-derived xenografts. Cell Rep..

[178] Nesic K (2021). Acquired RAD51C promoter methylation loss causes PARP inhibitor resistance in high-grade serous ovarian carcinoma. Cancer Res..

[179] Pauli C (2017). Personalized in vitro and in vivo cancer models to guide precision medicine. Cancer Discov..

[180] Yang M (2019). Afatinib treatment for her-2 amplified metastatic colorectal cancer based on patient-derived xenograft models and next generation sequencing. Cancer Biol. Ther..

[181] Coussy F (2020). Response to mTOR and PI3K inhibitors in enzalutamide-resistant luminal androgen receptor triple-negative breast cancer patient-derived xenografts. Theranostics.

[182] Arena S (2020). A subset of colorectal cancers with cross-sensitivity to olaparib and oxaliplatin. Clin. Cancer Res.

[183] Krumbach R (2011). Primary resistance to cetuximab in a panel of patient-derived tumour xenograft models: activation of MET as one mechanism for drug resistance. Eur. J. Cancer.

[184] Silic-Benussi M (2022). mTOR inhibition downregulates glucose-6-phosphate dehydrogenase and induces ROS-dependent death in T-cell acute lymphoblastic leukemia cells. Redox Biol..

[185] Zhang H (2020). Dynamic alterations of genome and transcriptome in KRAS G13D mutant CRC PDX model treated with cetuximab. BMC Cancer.

[186] Lupo B (2020). Colorectal cancer residual disease at maximal response to EGFR blockade displays a druggable Paneth cell-like phenotype. Sci. Transl. Med..

[187] Liu Z (2018). Mouse avatar models of esophageal squamous cell carcinoma proved the potential for EGFR-TKI afatinib and uncovered Src family kinases involved in acquired resistance. J. Hematol. Oncol..

[188] Li F (2022). Regulation of TORC1 by MAPK signaling determines sensitivity and acquired resistance to trametinib in pediatric BRAFV600E brain tumor models. Clin. Cancer Res..

[189] Zhao M (2021). Combining neratinib with CDK4/6, mTOR, and MEK inhibitors in models of HER2-positive cancer. Clin. Cancer Res..

[190] Fok JHL (2019). AZD7648 is a potent and selective DNA-PK inhibitor that enhances radiation, chemotherapy and olaparib activity. Nat. Commun..

[191] Liu ACH (2022). Targeting STAT5 signaling overcomes resistance to IDH inhibitors in acute myeloid leukemia through suppression of stemness. Cancer Res..

[192] Kavuri SM (2015). HER2 activating mutations are targets for colorectal cancer treatment. Cancer Discov..

[193] Gebreyohannes YK (2019). Robust activity of avapritinib, potent and highly selective inhibitor of mutated KIT, in patient-derived xenograft models of gastrointestinal stromal tumors. Clin. Cancer Res..

[194] Wang B (2021). Targeting mTOR signaling overcomes acquired resistance to combined BRAF and MEK inhibition in BRAF-mutant melanoma. Oncogene.

[195] Zheng Q (2021). A novel STAT3 inhibitor W2014-S regresses human non-small cell lung cancer xenografts and sensitizes EGFR-TKI acquired resistance. Theranostics.

[196] Ciardiello D (2019). Immunotherapy of colorectal cancer: challenges for therapeutic efficacy. Cancer Treat. Rev..

[197] Vilar E, Gruber SB (2010). Microsatellite instability in colorectal cancer-the stable evidence. Nat. Rev. Clin. Oncol..

[198] Kawazu M (2022). HLA class I analysis provides insight into the genetic and epigenetic background of immune evasion in colorectal cancer with high microsatellite instability. Gastroenterology.

[199] Ganesh K (2019). Immunotherapy in colorectal cancer: rationale, challenges and potential. Nat. Rev. Gastroenterol. Hepatol..

[200] Westcott PMK (2021). Low neoantigen expression and poor T-cell priming underlie early immune escape in colorectal cancer. Nat. Cancer.

[201] Lin A, Zhang J, Luo P (2020). Crosstalk between the MSI status and tumor microenvironment in colorectal cancer. Front. Immunol..

[202] Sanmamed MF (2021). A burned-out CD8(+) T-cell subset expands in the tumor microenvironment and curbs cancer immunotherapy. Cancer Discov..

[203] Ribas A (2016). PD-1 blockade expands intratumoral memory T cells. Cancer Immunol. Res..

[204] Tumeh PC (2014). PD-1 blockade induces responses by inhibiting adaptive immune resistance. Nature.

[205] Nguyen R (2019). Interleukin-15 enhances anti-GD2 antibody-mediated cytotoxicity in an orthotopic PDX model of neuroblastoma. Clin. Cancer Res..

[206] Wu HW (2019). Anti-CD105 antibody eliminates tumor microenvironment cells and enhances anti-GD2 antibody immunotherapy of neuroblastoma with activated natural killer cells. Clin. Cancer Res..

[207] Wang X (2019). TOX promotes the exhaustion of antitumor CD8(+) T cells by preventing PD1 degradation in hepatocellular carcinoma. J. Hepatol..

[208] Song X (2018). Self-supplied tumor oxygenation through separated liposomal delivery of H2O2 and catalase for enhanced radio-immunotherapy of cancer. Nano Lett..

[209] Ni W (2021). Targeting cholesterol biosynthesis promotes anti-tumor immunity by inhibiting long noncoding RNA SNHG29-mediated YAP activation. Mol. Ther..

[210] Wu Q (2022). Remodeling chondroitin-6-sulfate-mediated immune exclusion enhances anti-PD-1 response in colorectal cancer with microsatellite stability. Cancer Immunol. Res..

[211] Raj D (2019). Switchable CAR-T cells mediate remission in metastatic pancreatic ductal adenocarcinoma. Gut.

[212] Lee YH (2017). Inhibition of the B7-H3 immune checkpoint limits tumor growth by enhancing cytotoxic lymphocyte function. Cell Res..

[213] Du H (2019). Antitumor responses in the absence of toxicity in solid tumors by targeting B7-H3 via chimeric antigen receptor T cells. Cancer Cell.

[214] Fauci JM (2014). Monoclonal antibody-based immunotherapy of ovarian cancer: targeting ovarian cancer cells with the B7-H3-specific mAb 376.96. Gynecol. Oncol..

[215] Luther SA (2002). Differing activities of homeostatic chemokines CCL19, CCL21, and CXCL12 in lymphocyte and dendritic cell recruitment and lymphoid neogenesis. J. Immunol..

[216] Link A (2007). Fibroblastic reticular cells in lymph nodes regulate the homeostasis of naive T cells. Nat. Immunol..

[217] Goto S (2021). Enhanced anti-tumor efficacy of IL-7/CCL19-producing human CAR-T cells in orthotopic and patient-derived xenograft tumor models. Cancer Immunol. Immunother..

[218] Morsut L (2016). Engineering customized cell sensing and response behaviors using synthetic notch receptors. Cell.

[219] Choe JH (2021). SynNotch-CAR T cells overcome challenges of specificity, heterogeneity, and persistence in treating glioblastoma. Sci. Transl. Med..

[220] Cabo M (2021). CD137 costimulation counteracts TGFbeta inhibition of NK-cell antitumor function. Cancer Immunol. Res..

[221] Long AH (2015). 4-1BB costimulation ameliorates T cell exhaustion induced by tonic signaling of chimeric antigen receptors. Nat. Med..

[222] Brown CE (2018). Optimization of IL13Ralpha2-targeted chimeric antigen receptor T cells for improved anti-tumor efficacy against glioblastoma. Mol. Ther..

[223] Yu J (2018). Anti-GD2/4-1BB chimeric antigen receptor T cell therapy for the treatment of Chinese melanoma patients. J. Hematol. Oncol..

[224] Stenger D (2020). Endogenous TCR promotes in vivo persistence of CD19-CAR-T cells compared to a CRISPR/Cas9-mediated TCR knockout CAR. Blood.

[225] Lin, T. Y. et al. Novel potent anti-STEAP1 bispecific antibody to redirect T cells for cancer immunotherapy. *J. Immunother.Cancer*. **9** (2021). 10.1136/jitc-2021-003114 PMC8438958 34497115

[226] Chow KT, Gale M, Loo YM (2018). RIG-I and Other RNA Sensors in Antiviral Immunity. Annu Rev. Immunol..

[227] Ruzicka M (2020). RIG-I-based immunotherapy enhances survival in preclinical AML models and sensitizes AML cells to checkpoint blockade. Leukemia.

[228] Logtenberg MEW, Scheeren FA, Schumacher TN (2020). The CD47-SIRPalpha Immune Checkpoint. Immunity.

[229] Qin H (2021). Systematic preclinical evaluation of CD33-directed chimeric antigen receptor T cell immunotherapy for acute myeloid leukemia defines optimized construct design. J. Immunother. Cancer.

[230] Veillette A, Chen J (2018). SIRPalpha-CD47 Immune Checkpoint Blockade in Anticancer Therapy. Trends Immunol..

[231] Michaels AD (2018). CD47 Blockade as an Adjuvant Immunotherapy for Resectable Pancreatic Cancer. *Clin*. Cancer Res.

[232] Shenouda MM (2017). Ex vivo expanded natural killer cells from breast cancer patients and healthy donors are highly cytotoxic against breast cancer cell lines and patient-derived tumours. Breast Cancer Res.

[233] Haikala HM (2022). EGFR Inhibition Enhances the Cellular Uptake and Antitumor-Activity of the HER3 Antibody-Drug Conjugate HER3-DXd. Cancer Res.

[234] Gandullo-Sanchez L (2020). HER3 targeting with an antibody-drug conjugate bypasses resistance to anti-HER2 therapies. EMBO Mol. Med.

[235] Altunel E (2020). Development of a precision medicine pipeline to identify personalized treatments for colorectal cancer. BMC Cancer.

[236] Zhu Y (2018). miR-145 Antagonizes SNAI1-Mediated Stemness and Radiation Resistance in Colorectal Cancer. Mol. Ther..

[237] Yang H (2020). CircPTK2 (hsa_circ_0005273) as a novel therapeutic target for metastatic colorectal cancer. Mol. Cancer.

[238] Chen ZH (2019). Eukaryotic initiation factor 4A2 promotes experimental metastasis and oxaliplatin resistance in colorectal cancer. J. Exp. Clin. Cancer Res.

[239] Parkins CC (2021). Mechanically matching the rheological properties of brain tissue for drug-delivery in human glioblastoma models. Biomaterials.

[240] Xu J (2021). Co-delivery of 5-fluorouracil and miRNA-34a mimics by host-guest self-assembly nanocarriers for efficacious targeted therapy in colorectal cancer patient-derived tumor xenografts. Theranostics.

[241] Ruigrok EAM (2021). Extensive preclinical evaluation of lutetium-177-labeled PSMA-specific tracers for prostate cancer radionuclide therapy. Eur. J. Nucl. Med Mol. Imaging.

[242] Hu B (2020). Establishment of a hepatocellular carcinoma patient-derived xenograft platform and its application in biomarker identification. Int J. Cancer.

[243] Shin, H. Y. et al. Identification of Prognostic Markers of Gynecologic Cancers Utilizing Patient-Derived Xenograft Mouse Models. *Cancers (Basel)*. **14**, (2022). 10.3390/cancers14030829 PMC8834149 35159096

[244] Pham NA (2021). Patient-derived tumor xenograft and organoid models established from resected pancreatic, duodenal and biliary cancers. Sci. Rep..

[245] Kawashima N (2022). Comparison of clonal architecture between primary and immunodeficient mouse-engrafted acute myeloid leukemia cells. Nat. Commun..

[246] Moy RH (2022). Defining and Targeting Esophagogastric Cancer Genomic Subsets With Patient-Derived Xenografts. JCO Precis Oncol..

[247] Cybula M (2021). Patient-Derived Xenografts of High-Grade Serous Ovarian Cancer Subtype as a Powerful Tool in Pre-Clinical Research. Cancers.

[248] Peille AL (2020). Evaluation of molecular subtypes and clonal selection during establishment of patient-derived tumor xenografts from gastric adenocarcinoma. Commun. Biol..

[249] Ryu JS (2019). Integrative in vivo drug testing using gene expression signature and patient-derived xenografts from treatment-refractory HER2 positive and triple-negative subtypes of breast cancer. Cancers.

[250] Jo H (2022). Comparative study on the efficacy and exposure of molecular target agents in non-small cell lung cancer PDX models with driver genetic alterations. Mol. Cancer Ther..

[251] Xu W (2020). Comprehensive comparison of patient-derived xenograft models in Hepatocellular Carcinoma and metastatic Liver Cancer. Int. J. Med. Sci..

[252] Wu L (2017). Patient-derived xenograft establishment from human malignant pleural mesothelioma. Clin. Cancer Res..

[253] Struder D (2021). Establishment and characterization of patient-derived head and neck cancer models from surgical specimens and endoscopic biopsies. J. Exp. Clin. Cancer Res..

[254] Kamili A (2020). Accelerating development of high-risk neuroblastoma patient-derived xenograft models for preclinical testing and personalised therapy. Br. J. Cancer.

[255] Miyamoto S (2022). Validation of a Patient-Derived Xenograft Model for Cervical Cancer Based on Genomic and Phenotypic Characterization. Cancers.

[256] Tanaka K (2022). The first Japanese biobank of patient-derived pediatric acute lymphoblastic leukemia xenograft models. Cancer Sci..

[257] Tew BY (2020). Patient-derived xenografts of central nervous system metastasis reveal expansion of aggressive minor clones. Neuro Oncol..

[258] Chapuy B (2016). Diffuse large B-cell lymphoma patient-derived xenograft models capture the molecular and biological heterogeneity of the disease. Blood.

[259] Baschnagel AM (2021). Development and characterization of patient-derived xenografts from non-small cell lung cancer brain metastases. Sci. Rep..

[260] Elst L (2022). Establishment and characterization of advanced penile cancer patient-derived tumor xenografts: paving the way for personalized treatments. Eur. Urol. Focus.

[261] Lilja-Fischer JK (2019). Characterization and radiosensitivity of HPV-related oropharyngeal squamous cell carcinoma patient-derived xenografts. Acta Oncol..

[262] Zhang L (2017). B-cell lymphoma patient-derived xenograft models enable drug discovery and are a platform for personalized therapy. Clin. Cancer Res..

[263] Chamberlain CE (2018). A patient-derived xenograft model of pancreatic neuroendocrine tumors identifies sapanisertib as a possible new treatment for everolimus-resistant tumors. Mol. Cancer Ther..

[264] Lin CY (2021). A patient-derived xenograft model of dedifferentiated endometrial carcinoma: a proof-of-concept study for the identification of new molecularly informed treatment approaches. Cancers.

[265] Yagishita S (2021). Characterization of the large-scale Japanese patient-derived xenograft (J-PDX) library. Cancer Sci..

[266] Schueler J (2019). Induction of acquired resistance towards EGFR inhibitor gefitinib in a patient-derived xenograft model of non-small cell lung cancer and subsequent molecular characterization. Cells.

[267] Zhang T (2020). Discovery of a novel third-generation EGFR inhibitor and identification of a potential combination strategy to overcome resistance. Mol. Cancer.

[268] Chew NJ (2021). Evaluation of FGFR targeting in breast cancer through interrogation of patient-derived models. Breast Cancer Res..

[269] Krytska K (2016). Crizotinib synergizes with chemotherapy in preclinical models of neuroblastoma. Clin. Cancer Res..

[270] Shattuck-Brandt RL (2020). Metastatic melanoma patient-derived xenografts respond to MDM2 inhibition as a single agent or in combination with BRAF/MEK inhibition. Clin. Cancer Res..

[271] Kinsey CG (2019). Protective autophagy elicited by RAF->MEK->ERK inhibition suggests a treatment strategy for RAS-driven cancers. Nat. Med..

[272] Coussy F (2020). Combination of PI3K and MEK inhibitors yields durable remission in PDX models of PIK3CA-mutated metaplastic breast cancers. J. Hematol. Oncol..

[273] Hsu PY (2018). Dual mTOR kinase inhibitor MLN0128 sensitizes HR(+)/HER2(+) breast cancer patient-derived xenografts to trastuzumab or fulvestrant. Clin. Cancer Res..

[274] Harris FR (2019). Targeting HER2 in patient-derived xenograft ovarian cancer models sensitizes tumors to chemotherapy. Mol. Oncol..

[275] Hashimoto Y (2019). A novel HER3-targeting antibody-drug conjugate, U3-1402, exhibits potent therapeutic efficacy through the delivery of cytotoxic payload by efficient internalization. Clin. Cancer Res..

[276] Reddy TP (2020). Simultaneous targeting of HER family pro-survival signaling with Pan-HER antibody mixture is highly effective in TNBC: a preclinical trial with PDXs. Breast Cancer Res..

[277] Odintsov I (2021). Novel preclinical patient-derived lung cancer models reveal inhibition of HER3 and MTOR signaling as therapeutic strategies for NRG1 fusion-positive cancers. J. Thorac. Oncol..

[278] Chen L (2022). Discovery of novel KRAS‒PDEdelta inhibitors with potent activity in patient-derived human pancreatic tumor xenograft models. Acta Pharm. Sin. B.

[279] Barrette AM (2022). Anti-invasive efficacy and survival benefit of the YAP-TEAD inhibitor verteporfin in preclinical glioblastoma models. Neuro Oncol..

[280] Hemming ML (2022). Preclinical modeling of leiomyosarcoma identifies susceptibility to transcriptional CDK inhibitors through antagonism of E2F-driven oncogenic gene expression. Clin. Cancer Res..

[281] Karalis JD (2022). Lenvatinib inhibits the growth of gastric cancer patient-derived xenografts generated from a heterogeneous population. J. Transl. Med..

[282] Dankner M (2018). Dual MAPK inhibition is an effective therapeutic strategy for a subset of class II BRAF mutant melanomas. Clin. Cancer Res..

[283] Knudsen ES (2021). Targeting dual signalling pathways in concert with immune checkpoints for the treatment of pancreatic cancer. Gut.

[284] Gymnopoulos M (2020). TR1801-ADC: a highly potent cMet antibody-drug conjugate with high activity in patient-derived xenograft models of solid tumors. Mol. Oncol..

[285] Vaisitti T (2021). ROR1 targeting with the antibody-drug conjugate VLS-101 is effective in Richter syndrome patient-derived xenograft mouse models. Blood.

[286] Hallin J (2020). The KRAS(G12C) inhibitor MRTX849 provides insight toward therapeutic susceptibility of KRAS-mutant cancers in mouse models and patients. Cancer Discov..

[287] Song X (2020). Mcl-1 inhibition overcomes intrinsic and acquired regorafenib resistance in colorectal cancer. Theranostics.

[288] Liu C (2021). Pharmacological targeting PTK6 inhibits the JAK2/STAT3 sustained stemness and reverses chemoresistance of colorectal cancer. J. Exp. Clin. Cancer Res..

[289] Tan X (2019). BET inhibitors potentiate chemotherapy and killing of spop-mutant colon cancer cells via induction of DR5. Cancer Res..

[290] Shang J (2020). Small-molecule activating SIRT6 elicits therapeutic effects and synergistically promotes anti-tumor activity of vitamin D3 in colorectal cancer. Theranostics.

[291] Kurth I (2021). Therapeutic targeting of SLC6A8 creatine transporter suppresses colon cancer progression and modulates human creatine levels. Sci. Adv..

[292] Wagner J (2018). Anti-tumor effects of ONC201 in combination with VEGF-inhibitors significantly impacts colorectal cancer growth and survival in vivo through complementary non-overlapping mechanisms. J. Exp. Clin. Cancer Res..

[293] Li C (2021). Dehydrodiisoeugenol inhibits colorectal cancer growth by endoplasmic reticulum stress-induced autophagic pathways. J. Exp. Clin. Cancer Res..

[294] Hinze L (2020). Exploiting the therapeutic interaction of WNT pathway activation and asparaginase for colorectal cancer therapy. Cancer Discov..

[295] Villa A (2021). Transplantation of autologous extracellular vesicles for cancer-specific targeting. Theranostics.

[296] Chen J (2020). Spatiotemporally targeted nanomedicine overcomes hypoxia-induced drug resistance of tumor cells after disrupting neovasculature. Nano Lett..

[297] Lee KJ (2020). A novel nanoparticle-based theranostic agent targeting LRP-1 enhances the efficacy of neoadjuvant radiotherapy in colorectal cancer. Biomaterials.

[298] Liang C (2021). Pi electron-stabilized polymeric micelles potentiate docetaxel therapy in advanced-stage gastrointestinal cancer. Biomaterials.

